# Exposure to road traffic noise and cognitive development in schoolchildren in Barcelona, Spain: A population-based cohort study

**DOI:** 10.1371/journal.pmed.1004001

**Published:** 2022-06-02

**Authors:** Maria Foraster, Mikel Esnaola, Mónica López-Vicente, Ioar Rivas, Mar Álvarez-Pedrerol, Cecilia Persavento, Nuria Sebastian-Galles, Jesus Pujol, Payam Dadvand, Jordi Sunyer

**Affiliations:** 1 ISGlobal, Barcelona, Spain; 2 Universitat Pompeu Fabra (UPF), Barcelona, Spain; 3 CIBER Epidemiología y Salud Pública (CIBEREsp), Madrid, Spain; 4 PHAGEX Research Group, Blanquerna School of Health Science, Universitat Ramon Llull (URL), Barcelona, Spain; 5 Department of Child and Adolescent Psychiatry and Psychology, Erasmus MC University Medical Center, Rotterdam, the Netherlands; 6 MRI Research Unit, Department of Radiology, Hospital del Mar, Barcelona, Spain; 7 Centro Investigación Biomédica en Red de Salud Mental, CIBERSAM, Barcelona, Spain; 8 IMIM (Hospital del Mar Medical Research Institute), Barcelona, Spain; Wolfson Institute of Preventive Medicine, UNITED KINGDOM

## Abstract

**Background:**

Road traffic noise is a prevalent and known health hazard. However, little is known yet about its effect on children’s cognition. We aimed to study the association between exposure to road traffic noise and the development of working memory and attention in primary school children, considering school-outdoor and school-indoor annual average noise levels and noise fluctuation characteristics, as well as home-outdoor noise exposure.

**Methods and findings:**

We followed up a population-based sample of 2,680 children aged 7 to 10 years from 38 schools in Barcelona (Catalonia, Spain) between January 2012 to March 2013. Children underwent computerised cognitive tests 4 times (*n* = 10,112), for working memory (2-back task, detectability), complex working memory (3-back task, detectability), and inattentiveness (Attention Network Task, hit reaction time standard error, in milliseconds). Road traffic noise was measured indoors and outdoors at schools, at the start of the school year, using standard protocols to obtain A-weighted equivalent sound pressure levels, i.e., annual average levels scaled to human hearing, for the daytime (daytime LAeq, in dB). We also derived fluctuation indicators out of the measurements (noise intermittency ratio, %; and number of noise events) and obtained individual estimated indoor noise levels (LAeq) correcting for classroom orientation and classroom change between years. Home-outdoor noise exposure at home (Lden, i.e., EU indicator for the 24-hour annual average levels) was estimated using Barcelona’s noise map for year 2012, according to the European Noise Directive (2002). We used linear mixed models to evaluate the association between exposure to noise and cognitive development adjusting for age, sex, maternal education, socioeconomical vulnerability index at home, indoor or outdoor traffic-related air pollution (TRAP) for corresponding school models or outdoor nitrogen dioxide (NO_2_) for home models. Child and school were included as nested random effects.

The median age (percentile 25, percentile 75) of children in visit 1 was 8.5 (7.8; 9.3) years, 49.9% were girls, and 50% of the schools were public. School-outdoor exposure to road traffic noise was associated with a slower development in working memory (2-back and 3-back) and greater inattentiveness over 1 year in children, both for the average noise level (e.g., ‒4.83 points [95% CI: ‒7.21, ‒2.45], *p*-value < 0.001, in 2-back detectability per 5 dB in street levels) and noise fluctuation (e.g., ‒4.38 [‒7.08, ‒1.67], *p*-value = 0.002, per 50 noise events at street level). Individual exposure to the road traffic average noise level in classrooms was only associated with inattentiveness (2.49 ms [0, 4.81], *p*-value = 0.050, per 5 dB), whereas indoor noise fluctuation was consistently associated with all outcomes. Home-outdoor noise exposure was not associated with the outcomes. Study limitations include a potential lack of generalizability (58% of mothers with university degree in our study versus 50% in the region) and the lack of past noise exposure assessment.

**Conclusions:**

We observed that exposure to road traffic noise at school, but not at home, was associated with slower development of working memory, complex working memory, and attention in schoolchildren over 1 year. Associations with noise fluctuation indicators were more evident than with average noise levels in classrooms.

## Abstract 2

In a cohort study, Maria Foraster and colleagues study associations between exposure to road traffic noise at schools, and trajectories of working memory and inattentiveness among schoolchildren aged 7 to 10 years in Barcelona, Spain.

## Author summary

**Why was this study done?:**

- Exposure to aircraft noise has been associated with impaired cognitive development in schoolchildren, and experiments have also observed that animals exposed to moderate or high noise levels for 4 to 30 days suffer changes in the brain.
- Road traffic noise is the most common noise source and many children are exposed to it at school; however, it is still unclear whether it affects children’s cognitive development, including important aspects such as working memory or attention.
- Moreover, while previous studies have assessed exposure to the average noise level outside the school, none have assessed whether the noise peaks of fluctuating traffic and exposure to noise inside the classroom could affect children’s cognition.

**What did the researchers do and find?:**

- We carried out a cohort study to assess whether school exposure (inside and outside classrooms) and home exposure to road traffic noise were associated with the development of working memory and attention over 12 months in 2,680 children aged 7 to 10 years from 38 schools in Barcelona, Spain.
- We assessed long-term exposure to road traffic noise outside and inside the school with measurements (average noise levels and noise fluctuation) and at home with Barcelona’s noise map (outdoor average noise levels).
- We used computerised tests every 3 months over 12 months to measure the development of working memory (the system that keeps and manipulates transitory information), complex working memory (it further involves continuous updating of the working memory), and inattentiveness in children.
- We observed that higher exposure to road traffic noise outside and inside the school, but not at home, was associated with a slower development of working memory and a slower improvement of inattentiveness over 12 months. Inside the classroom, associations were more evident for exposure to noise fluctuation than to average noise levels.

**What do these findings mean?:**

- These findings suggest that, in children aged 7 to 10 years in Barcelona, higher exposure to road traffic noise at school relates to poorer development of attention and working memory. These are important for learning.
- The findings might not be applicable to other populations and need replication in other locations.
- These findings are of public health relevance given the large number of children exposed to road traffic noise in schools and support the implementation of environmental noise policies that protect the school environment.

## Introduction

Road traffic noise is the most prevalent environmental and transportation noise source in Europe [1]. Transportation noise is the second most detrimental environmental factor for ill health in Europe, just after air pollution [2,3]. Such health impact is supported by an increasing number of epidemiological studies in adults; however, little is known yet about the effects in children [4–6].

One of the first adverse effects of noise on children could relate to cognitive development, given that childhood is a vulnerable period for brain maturation [7]. It is suggested that noise may impact cognitive abilities directly or lead to impaired attention, frustration, learned helplessness, arousal, or tuning out, which could impact performance and learning in the long term [8]. The impact of noise on cognition is supported by animal experiments in rats in which subchronic exposure to white noise (4 hours/day, 100 dB, up to 30 days) led to electroencephalographic changes in the occipital and prefrontal regions (relevant for executive functions) [9,10] and to reduced dendrite number in the hippocampus (relevant for learning and memory) [10]. An experiment exposing mice to average noise levels closer to environmental noise levels (72 dB continuous aircraft noise, up to 4 days) further observed increased oxidative stress in the brain, particularly in the frontal cortex [11].

In schoolchildren, there is substantial evidence for the association between exposure to aircraft noise and decreased cognitive development, particularly for reading comprehension, achievement tests, and long-term memory, according to longitudinal studies, intervention studies, and recent systematic reviews [5,8]. However, the available evidence for the association between exposure to road traffic noise and children’s cognition is still limited and based only on cross-sectional studies [5,8]. In specific, it is unclear how noise could affect working memory or attention, which are essential for learning and school attainment [12,13] and which develop actively between 6 to 10 years of age as a result of cognitive maturation [14–16]. To our knowledge, only 5 cross-sectional studies from 3 different projects have examined the association between exposure to road traffic noise and tests of attention and they used different tests yielding mixed results [17–21]. Another 5, all part of the same cross-sectional RANCH study (road traffic and aircraft noise exposure and children’s cognition and health), evaluated the association between exposure to road traffic noise and tests of working memory and reported no effects [19–23]. All of these studies except Cohen and colleagues [17], evaluated school-outdoor road traffic noise levels, using measured or modelled A-weighted equivalent noise levels (LAeq), which were representative of annual average noise levels. Cohen and colleagues [17] only assessed home-outdoor road traffic noise exposure, but using floor level as a proxy of noise exposure. Van Kempen and colleagues [20,21] assessed both school-outdoor and home-outdoor average levels of road traffic noise, and only observed associations between school-outdoor noise exposure and worse attention.

Importantly, none of the few previous studies have evaluated indoor road traffic noise levels in the classroom, where repeated exposure to noise could affect concentration, learning, and cognition [7]. Moreover, studies have focused on exposure to average noise levels, whereas the role of noise fluctuation (i.e., the presence of peaks and noise intermittency) on children’s cognition remains unknown. Indirect evidence in adults indicates an association of noise fluctuation with endothelial dysfunction [24] and higher annoyance in people exposed to greater road traffic noise intermittency [25]. Finally, only 2 RANCH studies adjusted associations for coexposure to traffic-related air pollution [21,22], which has been also related with cognitive impairment in children [26]. In conclusion, there is a need for longitudinal studies and of novel, comprehensive assessments of children’s exposure to noise at school and home in order to understand the effects of exposure to road traffic noise on children’s cognitive development.

We hypothesise that school and home exposure to road traffic noise impairs the development of working memory and attention in children. The aim of the present study was to assess the association between exposure to road traffic noise indoors and outdoors at schools, accounting both for the annual average noise level and the noise fluctuation characteristics, with the development of working memory and inattentiveness over 12 months in primary schoolchildren, based on the BREATHE project (Brain Development and Air Pollution Ultrafine Particles in School Children), a cohort study of children in Barcelona. The study also assessed the association between home-outdoor long-term exposure to road traffic noise with the development of working memory and inattentiveness.

## Methodology

### Study population and design

This longitudinal study was carried out in the city of Barcelona, within the context of the BREATHE project (2011 to 2016). The BREATHE project aimed to study how air pollutants, including noise pollution, affected children’s cognition. Barcelona is a densely populated city in northeast of Spain, in which the main source of noise is road traffic noise. In Barcelona, a total of 53% of the population are exposed to road traffic noise levels above the recommended levels by the World Health Organization (24-hour EU noise indicator: Lden ≥ 53 dB) [4,27]. The BREATHE project assessed 2,897 children aged 7 to 10 years who were followed up through 2 academic courses between years 2012 and 2013 with a total of 4 visits and who attended 39 schools in Barcelona (Catalonia, Spain). Schools were selected based on the range of estimated outdoor levels of traffic-related nitrogen dioxide (NO_2_) [28] in Barcelona, which would therefore also represent the range of noise as another traffic-related factor. We paired schools with low and high NO_2_ levels by socioeconomic vulnerability index (SES) and type of school (i.e., private/public). In specific, we identified schools in Barcelona with low to moderate NO_2_ levels (maximum 51 μg/m^3^, mean of the city) estimated with a land use regression model [28] and searched schools with higher NO_2_ levels but of similar type and SES (S1 Table). The participating schools were representative of the rest of schools in Barcelona in terms of neighbourhood socioeconomic vulnerability index (0.46 versus 0.50, Kruskal–Wallis test, *p*-value = 0.570) and NO_2_ levels (51.5 versus 50.9 μg/m^3^, Kruskal–Wallis test, *p*-value = 0.720). We invited all children from second to fourth grade who had no special needs and a 59% of families agreed to participate. All children had attended the same school for at least 6 months and a 98% for more than 1 year. In the present study, we excluded 1 school due to incomplete noise data. This study did not have a prespecified analysis plan.

All the participating children’s parents or guardians signed and provided the informed consent. The research procedures were explained in detail to the children, who could ask any question. All included children provided their assent. The study received ethical approval by the Clinical Research Ethical Committee (No. 2010/41221/I) of the Institut Hospital del Mar d’Investigacions Mèdiques–Parc de Salut Mar, Barcelona, Spain, and the FP7-ERC-2010-AdG Ethics Review Committee.

This study is reported as per the Strengthening the Reporting of Observational Studies in Epidemiology (STROBE) guideline (S1 STROBE Checklist).

### Noise exposure assessment

#### Measured average noise levels at school

Environmental campaigns started in January 2012 and were performed twice, 6 months apart, during the cold and hot seasons. School pairs were assessed simultaneously. Road traffic noise was measured indoors (in classrooms) in the first campaign and indoors/outdoors 6 months later during the second campaign, following ISO 1996–2 (2007) for long-term environmental noise assessment based on short-term measurements. As part of this protocol, we carried out supervised time-stamped 30-min recordings of A-weighted equivalent noise levels (LAeq, in dB) during 2 consecutive weekdays before lessons started, using a calibrated CESVA SC160 device (type II microphone). We took simultaneous measurements indoors in 1 classroom (LAeq,in) and outdoors in the playground (LAeq,playgr.), followed right after by a street recording in front of the school (LAeq,street). Windows were closed during the measurement. Noise artefacts were subtracted to obtain clean road noise measurements. The 2-day mean of the measurements at each location during the second campaign, which assessed all school environments, was used as the exposure estimate.

Postprocessing analysis further confirmed the validity of the short-term noise protocol (ISO 1996–2) and the use of the 2-day mean to represent long-term (i.e., annual) means. Indoor noise measurements exhibited high reproducibility, i.e., intraclass correlation between the 2-day mean of each campaign = 0.89 and of the mean of the 2 campaigns = 0.94 (*p*-values < 0.001, 1-way random effects model). Moreover, the LAeq, street average was highly correlated (Spearman’s rank r = 0.86, Pearsons’ r = 0.85, *p*-values < 0.001) with the annual average noise levels for the daytime (Lday) obtained at the same location from the 2012 Strategic Noise Map for Barcelona derived under the EU Directive 2002/49/EC [29]. The noise map was published in year 2012 and represents average noise levels before year 2012.

To provide individual indoor average noise levels for each child during the study period (LAeq,in-indiv.), we further modelled indoor average levels of road traffic noise for all classrooms (adjusted R^2^ = 67.7%; see S1 Text), based on data on the floor level of classrooms, room orientation (classroom oriented towards: indoor area, outdoor courtyard, or directly to the street), outdoor levels of road traffic noise, the type of courtyard (open, semi-open, built), and the type of windows, among others, and accounted for child’s change of classroom between school years.

#### Measured noise fluctuation at school

We calculated the average number of individual noise events (NE) at each of the measured locations, i.e., street (NE, street), playground (NE, playgr.), and indoors (NE, in) as defined by [30] and previously used [24]. A noise event was labelled as a noise peak whose maximum noise level exceeded 3 dB above the total LAeq level during the measurement period [30]. Subsequently, noise intermittency ratio at the measured locations (IR, street; IR, playgr.; and IR, in) was calculated as the ratio between the LAeq noise level of the noise events for period T (Leq,T,events) and the total LAeq noise level for the same period T (Leq,T,tot), both expressed in sound energy (unit: percent) (Eq 1).


$$IR=\frac{10^{0.1Leq,T,events}}{10^{0.1Leq,T,tot}}x\;100$$ (1)

These fluctuation metrics were designed by Wunderli and colleagues [30], after the original planning of the current study. These metrics were calculated from the original noise measurement data.

#### Modelled average noise levels at school and home

We assigned exposure to road traffic noise as annual average road traffic noise levels at the geocoded noise measurement location in front of the school and at the geocoded home address of each participant, using the 2012 Strategic Noise Map of Barcelona, derived under the European Directive 2002/EC/49 [29]. Assignment of modelled noise levels was only possible in the 34 schools and 2,346 home addresses that fell within the boundaries of the city noise map, which excludes the outskirts of the city where 4 schools and some children’s homes were located. Specifically, at schools, we estimated the A-weighted equivalent noise levels for the daytime (Lday, from 7 AM to 9 PM) to account for children’s exposure during the academic time window. At home, we estimated the standard EU indicator for the 24 hours, i.e., the A-weighted equivalent noise levels for the 24 hours (Lden) with 5 dB and 10 dB penalties for the evening (9 PM to 11 PM) and nighttime (11 PM to 7 AM) to account for the children’s exposure during the nonacademic hours, weekends, and holidays during the year.

### Outcomes

From January 2012 to March 2013, children were visited 4 times (every 3 months) to carry out computerised psychometric measurements for working memory and inattentiveness in sessions of approximately 40 minutes. Tests were performed at schools by trained fieldworkers, who noted any incidents (including noise in the room) during the test. All children wore earphones to perform the tasks, which limited any influence of external noise stimuli on their outcomes.

We assessed 2 cognitive functions that develop rapidly during preadolescence, namely: working memory, assessed through the *n*-back task [14,31] and inattentiveness, assessed through the Attentional Network Test (ANT) [32,33]. Both tests have been validated as measures of neuropsychological development [32,34,35].

Working memory and attention are essential for learning and school attainment [12,13]. Working memory functions permit the maintenance and manipulation of information over short periods of time, and complex working memory requires continued, effective processing of information held in working memory stores. It underlies many other aspects of cognition, including learning, problem solving, reasoning, mathematics, and language comprehension [31]. Attention includes processes such as selectively attending to specific stimuli, focusing for prolonged periods on a task or incoming stimuli, or regulating and monitoring actions [14,36].

The experimental tasks were created for the project using the psychology experiment computer program E-Prime version 2.0 (Psychology Software Tools) and have been previously described [34,36,37].

In the *n*-back task, children observed a series of stimuli presented in the centre of the laptop’s screen, and they were instructed to press a specific keyboard button whenever a given stimulus was the same as the one presented n trials previously (1-, 2-, and 3-back). Participants completed 3 blocks (1-, 2-, and 3-back) for each stimulus. Each block consisted of 25 trials, and blocks were separated by a short break (5 to 20 seconds). The first 3 trials of each block were never a target, and 33% of stimuli of the following trials were targets. The completion of a target was followed by a motivational sound (“woo hoo!”) and a smiling face [37].

In the ANT, the screen showed a row of 5 yellow fish appearing either above or below a fixation point. Children were invited to “feed” the central fish as quickly as possible by pressing either the right or the left arrow key depending on the direction in which the target fish was pointing while ignoring the flanker fish, which pointed in either the same (congruent) or opposite (incongruent) direction than the middle fish. Visual signals informed about the approach of the target only (alerting cue) or about the approach of the target as well as its location (orienting cue) [38]. Each correct answer was followed by a simple animation sequence (the target fish blowing bubbles) and a recorded sound (“woo hoo!”). Incorrect responses were followed by a single tone and no animation of the fish [32]. A session consisted of 16 practice trials and 4 experimental blocks, each with 32 trials (128 trials in total) [36].

For the current study, we selected the following specific indicators because they showed little learning effect and an incremental growth in the repeated measurements during the study period. For the *n*-back test, we selected 2 loads (2-back and 3-back) and the numbers stimuli [26]. The 2-back test (or working memory hereafter) predicts general mental abilities, whereas the 3-back test (or complex working memory hereafter) is more complex to perform for children and could predict superior functions such as fluid intelligence [39]. All sets of *n*-back tests started with colours as a training phase and followed by the number stimuli. We evaluated the *n*-back parameter *d* prime (*d′*), a measure of detectability, obtained by subtracting the normalised false alarm rate from the hit rate: (*Z*_hit rate_−*Z*_false alarm rate_) × 100. A higher *d′* indicates more accurate test performance, i.e., better working memory or complex working memory performance. For the ANT, we selected the hit reaction time standard error (HRT-SE). This is a measure of response speed consistency for correct responses throughout the test [40]. As such, a higher HRT-SE indicates a highly variable reaction, thus more inattentiveness (i.e., poorer sustained attention).

### Covariates

We collected questionnaire information from parents about maternal and paternal education, marital status, occupation, family origin, gestational age at delivery, birth weight of the child, smoking during pregnancy, breastfeeding, siblings, adoption, and use of computer games. Area-level socioeconomic position (SES) at school and at home was derived using the neighbourhood socioeconomic vulnerability index (area-level SES) at census tract level (median area of 0.08 km^2^), a combined measure of 21 indicators covering 4 main dimensions: socioeconomic vulnerability, sociodemographic vulnerability, housing vulnerability, and subjective perception of vulnerability [41]. We measured height and weight and defined overweight and obesity using standard procedures [42]. We also collected information about the type of school (public/private) and assessed school educational quality as the sum of the school level (low, middle, high score) obtained in the basic competences of languages (Spanish and Catalan) and maths in the 2010/2011 Programme for International Student Assessment [43], which was self-reported by schools. Parents also completed the Strengths and Difficulties Questionnaire (SDQ) on child behavioural problems [44].

We accounted for annual average traffic-related air pollution (TRAP) levels outdoors and indoors at school by deriving a TRAP index consisting of 2 main traffic-related air pollutants in Barcelona, namely elemental carbon (EC) and NO_2_ [45–47]. In parallel to noise measurements, these air pollutants were measured simultaneously indoors (in the classroom) and outdoors (in the courtyard) for each school pair. Measurements were carried out over two 1-week campaigns, which corresponded to the cold and warm seasons. EC was obtained with thermal-optical analyses [45] of the particulate matter <2.5 μm filters (high volume samples, quartz microfiber filters for sampling) deployed from 9 AM to 5 PM. Weekly NO_2_ concentrations were measured with passive NO_2_ samplers (NO_2_ diffusion tube, Gradko International, United Kingdom). Annual concentrations were obtained by averaging the two 1-week measures after temporal adjustment by the ratio of the annual average to the weekly concentrations measured at a fixed background air quality monitoring station in Barcelona [47]. Individual exposure to traffic-related air pollution at home was estimated as annual average NO_2_ levels at the geocoded postal addresses using land use regression models for the pollutants, as explained elsewhere [28].

### Statistical analyses

We included a total of 2,680 (92.5%) children with complete data (i.e., with information at least for 1 outcome and school noise exposure and with data on age, sex, maternal education, and TRAP exposure), representing 9,984 (93.6%) tests. To account for the multilevel nature of the data, we used linear mixed effects models with the 4 repeated cognitive measures as outcomes (for each 2-back, 3-back, and HRT-SE), each noise exposure variable (one for each separate model) as a fixed effect predictor and child and school as nested random effects. To model the changes in the 12-month cognitive development associated with noise exposure, we further included an interaction term between age and the studied noise exposure variable [26]. As part of this model, we also reported the baseline effect (visit 1), namely the cross-sectional association between noise exposure and the cognitive outcome before the evaluation of the 12-month change in cognitive development. All models were further adjusted for potential confounders, as previously used [26]: age (years), sex (girl versus boy), maternal education (none/primary/secondary/university) as an indicator of household SES, urban vulnerability index as an indicator of residential neighbourhood-level SES, and outdoor or indoor TRAPs for models using outdoor or indoor noise at school, respectively, and for NO_2_ at home for models using noise at home.

Moreover, we carried out stratified analyses to evaluate whether the trajectories of the cognitive outcomes over 1 year differed in children attending schools with low and high outdoor and indoor road traffic noise levels, using a cutoff of 55 dB for low/high outdoor noise levels, according to the EU definitions [1], and a cutoff of 30 dB for indoor noise according to the WHO guidelines for classroom noise levels [48]. The stratified models were adjusted for the same covariates of the linear mixed effects models described in the previous paragraph.

### Sensitivity analyses

To assess the robustness of the evaluated associations to residual confounding, we further adjusted the main models for (one at a time) type of school (public versus private), paternal education (none/primary/secondary/university), foreign origin (child or the 2 parents born outside Spain/the child and at least 1 parent born in Spain), marital status (married or stable couple/others), overweight (non-overweight: body mass index < 85th percentile/overweight or obese: body mass index ≥ 85th percentile of WHO definition) [42], computer games during weekends (2 hours or less/>2 hours), siblings (Yes/No), adoption (Yes/No), smoking during pregnancy (Yes/No), preterm birth (<37 weeks/≥37 weeks), birth weight (<2.5 kg/≥2.5 kg), breastfeeding (Yes/No), socioeconomical vulnerability index at school (range: 0 to 1), school education quality (range: 0 to 6), and behavioural problems (range: 0 to 32). We also controlled for the paired design by including the school pair as a random effect (upon request during peer review). We also adjusted the studied associations at school for exposure to road traffic noise at home in the subsample living within the geographical limits covered by the 2012 Strategic Noise Map of Barcelona (*n* = 2346).

To compare our outdoor noise results with previous studies, which used modelled noise levels at street level instead of measurements, and to further assess the representativeness of our measurements for long-term exposure (i.e., annual averages), we repeated the main analyses by replacing the measured noise indicator (LAeq, street) by the modelled annual average level of road traffic noise for the daytime (Lday) estimated at the same location (*n* = 34 schools).

We reported the estimated change in the evaluated outcomes for a 5 dB, 50 events, and 10% increase in the noise level, number of noise events, and intermittency ratio, respectively. The statistical significance level used was *p* < 0.05, 2-sided. Analyses were performed with R statistical package (Version 3.4.2, R Foundation for Statistical Computing, Vienna, Austria) and Stata (Release 14. College Station, TX: StataCorp LP, United States).

## Results

As shown in Table 1, a total of 2,508, 2,563, 2,493, and 2,420 children participated in visits 1 to 4, respectively, which represented a total participation of 2,680 children and 9,984 repeated outcome measurements. The median (percentile 25, percentile 75: p25; p75) age of the children in visit 1 was 8.5 (7.8; 9.3) years, and in visit 4, it was 9.4 (8.7; 10.2) years. During the 12-month period of the study, children’s median (p25; p75) working memory (detectability) increased from 221 (131; 363) to 263 (153; 392) points, complex working memory (detectability) from 112 (59; 171) to 129 (64; 212) points, and inattentiveness (HRT-SE) decreased from 267 (201; 337) to 223 (162; 291) milliseconds.

**Table 1:** Median (percentiles 25 and 75) of children’s age and cognitive outcomes (working memory and inattentiveness) at each of the 4 repeated visits.

Visit	*n*	Age	2-back numbers (*d′*)^a^	3-back numbers (*d′*)^b^	ANT HRT-SE (ms)^c^
		p50 (p25, p75)	p50 (p25, p75)	p50 (p25, p75)	p50 (p25, p75)
1	2,508	8.5 (7.8, 9.3)	221 (131, 363)	112 (59, 171)	267 (201, 337)
2	2,563	8.7 (8.0, 9.5)	221 (131, 392)	123 (59, 190)	248 (184, 317)
3	2,493	9.1 (8.4, 9.8)	235 (131, 392)	129 (59, 190)	243 (181, 315)
4	2,420	9.4 (8.7, 10.2)	263 (153, 392)	129 (64, 212)	223 (162, 291)

^a^Working memory: 2-back number stimuli (d’).

^b^Complex working memory: 3-back number stimuli (*d′*). *d′*: detectability, a higher value indicates better working memory.

^c^Inattentiveness: HRT-SE (ms) of the Attention Network Task, a higher value indicates greater inattentiveness.

ANT, Attentional Network Test; HRT-SE, hit reaction time standard error.

The school- and individual-level characteristics of the study sample are presented in Table 2. Among others, a total of 49.0% of the sample were girls, 58.7% of the children had mothers, and 53% had fathers with high educational level (i.e., university) and the mean (standard deviation: SD) neighbourhood socioeconomic vulnerability index at residential level was 0.4 (0.2) points. Out of the 38 participating schools, 50% were public schools. Schools’ mean (SD) road traffic noise levels were the highest in the street (LAeq,street), and decreased in the playground (LAeq,playgr) and even further indoors (LAeq,in) and for individual exposure in the child’s classroom (LAeq,in-indiv), respectively: 63.6 (6.3) dB, 53.5 (5.4) dB, 38.6 (5.2) dB, and 37.5 (4.1) dB. Schools’ mean (SD) noise intermittency ratio in the street (IR, street) was higher than in the playground (IR, playgr) and indoors (IR, in), respectively: 52.8 (16.2) %, 20.8 (11.3) %, and 25.0 (13.2) %. Similarly, the mean (SD) total number of noise events in the street (NE, street) was greater than in the playground (NE, playgr) and indoors (NE, in), respectively: 178.1 (59.6), 92.7 (49.0), and 102.6 (58.4) events. The same patterns were observed for school-outdoor versus school-indoor air pollution levels of EC, respectively: 1.5 (0.7) μg/m^3^ and 1.4 (0.6) μg/m^3^ and NO_2_, respectively: 48.2 (13.2) μg/m^3^ and 31.6 (13.1) μg/m^3^. School’s mean (SD) road traffic noise levels based on modelled estimates in the street was 65.6 (6.5) dB. Home-outdoor mean (SD) exposure to average road traffic noise (Lden) was 63.8 (7.8) dB and to NO_2_ it was 54.6 (17.9) μg/m^3^. Bivariate analyses (S2 Table) stratified by the median of LAeq,street at schools showed no statistically significant differences across percentage of public schools, neighbourhood socioeconomic vulnerability index at school or working and complex working memory at baseline. As main differences, schools with LAeq,street below the median (<63.5 dB) compared to those equal or above the median had less children with inattentiveness at visit 1 [median (interquartile range, IQR) = 262.3 (134.5) versus 271.9 (142.1), *p*-value < 0.012, Kruskal–Wallis test] and behavioural problems [median (IQR) = 7.0 (7.0) versus 8.0 (7.0), *p*-value < 0.001, Kruskal–Wallis test], had more girls (62.9% versus 54.1%, *p*-value < 0.001, χ^2^ test), schools with higher educational quality [median (IQR) = 5.0 (3.0) versus 3.0 (2.0), *p*-value < 0.001, Kruskal–Wallis test], mothers with higher educational level (57.1% versus 48.7%, < 0.001, χ^2^ test) and similarly for partners, and lower neighbourhood socioeconomic vulnerability index at home [median (IQR) = 0.4 (0.4) versus 0.5 (0.3), *p*-value < 0.001, Kruskal–Wallis test].

**Table 2:** School- and individual-level characteristics of the study sample.

Variables	Mean (SD) or %
**School-level variables (*n* = 38)**	
Type of school, public	50.0%
School socioeconomic vulnerability index (*n*)	0.5 (0.2)
School education quality (PISA 2012, *n*)	3.9 (1.6)
Outdoor average noise level (LAeq, dB), street	63.6 (6.3)
Outdoor average noise level (LAeq, dB), playground	53.5 (5.4)
Indoor average noise level (LAeq, dB)	38.6 (5.2)
Outdoor noise intermittency ratio (IR, %), street	52.8 (16.2)
Outdoor noise intermittency ratio (IR, %), playground	20.8 (11.3)
Indoor noise intermittency ratio (IR, %)	25.0 (13.2)
Outdoor noise events (NE, *n*), street	178.1 (59.6)
Outdoor noise events (NE, *n*), playground	92.7 (49.0)
Indoor noise events (NE, *n*)	102.6 (58.4)
Outdoor elemental carbon level (EC, μg/m^3^)	1.5 (0.7)
Indoor elemental carbon level (EC, μg/m^3^)	1.4 (0.6)
Outdoor nitrogen dioxide level (NO_2_, μg/m^3^)	48.2 (13.2)
Indoor nitrogen dioxide level (NO_2_, μg/m^3^)	31.6 (13.1)
Outdoor average noise level (Lday, dB) (modelled)^a^	65.6 (6.5)
**Individual-level variables (*n* = 2,680)**	
Age (years)	8.5 (1.4)
Girls	49.9%
Maternal education, university	58.7%
Paternal education, university	53.3%
Foreign origin (non-Spanish)	14.9%
Marital status, married	85%
Overweight, Yes	27.6%
Computer games weekend, >1 hour	70.4%
Siblings, Yes	79.0%
Adopted child, Yes	3.9%
Smoking during pregnancy, Yes	10.2%
Birth ≥ 37 weeks	92.1%
Birth weight ≥ 2.5 kg	90.0%
Breastfeeding, Yes	82.0%
Behavioural problems (SDQ)	8.4 (5.2)
Home socioeconomic vulnerability index (*n*)	0.4 (0.2)
Individual indoor average noise level in classroom (LAeq, dB)	37.5 (4.1)
Home-outdoor average noise level (Lden, dB) (modelled)^b^	63.8 (7.8)
Home-outdoor average NO_2_ level (μg/m^3^) (modelled)	54.6 (17.9)

^a^*n* = 34.

^b^*n* = 2,346.

Data are mean (standard deviation) or percentage (%).

LAeq: A-weighted equivalent noise levels; Lday: LAeq for the daytime (7 AM to 9 PM); Lden: LAeq for the 24 hours with 5 dB and 10 dB penalties for the evening (9 PM to 11 PM) and nighttime (11 PM to 7 AM), respectively; PISA: Programme for International Student Assessment; SDQ: Strengths and Difficulties Questionnaire.

Pearson correlations between school-noise indicators were generally low or moderate (S3 Table), with some exceptions of high correlations between outdoor average noise levels (LAeq,street versus LAeq,playgr, r = 0.74, *p*-value < 0.001), between playground indicators of intermittency ratio and number of noise events (IR,playgr versus NE,playgr, r = 0.82, *p*-value < 0.001), and between indoor indicators of intermittency ratio and number of noise events (IR,in versus NE,in, r = 0.83, *p*-value < 0.001). Correlations between street and playground (i.e., outdoor) noise indicators were higher than between outdoor and indoor noise indicators [r range (*p*-value), respectively = 0.32 (0.059) to 0.74 (<0.001) versus 0.14 (0.426) to 0.44 (<0.001)], except for IR,street which exhibited, overall, low correlation with other indicators. Regarding air pollution, the correlation between outdoor and indoor TRAPs was high (r = 0.79, *p*-value < 0.001). The correlation between outdoor TRAP and outdoor noise indicators at school was moderately high with LAeq [r range (*p*-value): r = 0.68 (<0.001) to 0.70 (<0.001)], moderate with NE [r range (*p*-value): 0.40 (0.014) to 0.41 (0.012)] and low with IR [r range (*p*-value): ‒0.15 (0.384) to 0.28 (0.094)]. Similar magnitudes were observed for the correlation between indoor TRAP and indoor noise indicators in the classroom. The correlation between home-outdoor average noise levels (Lden) and NO_2_ was r = 0.33 (*p*-value < 0.001). Finally, there was no correlation between outdoor exposure to noise at school and at home for any of the noise indicators [r range (*p*-value): ‒0.03 (0.165) to 0.02 (0.287)].

### Association of exposure to road traffic noise with working memory outcomes and inattentiveness

The adjusted models for the association between exposure to road traffic noise at school and home and working memory, complex working memory and inattentiveness are shown in Table 3. Unadjusted models are shown in S4 Table.

**Table 3:** Estimated effect (β) and 95% confidence intervals (95% CI) in working memory outcomes and inattentiveness at baseline and their 12-month change in association to school and home exposure to road traffic noise (*n* = 2,680 children, 9,984 repeats).

Road traffic noise indicators	Working memory (2-back numbers, *d’*)				Complex working memory (3-back numbers, *d’*)				Inattentiveness (Attention Network Task, HRT-SE[ms])			
	Baseline, β (95% CI)	*p*-value	12-month change, β (95% CI)	*p*-value	Baseline, β (95% CI)	*p*-value	12-month change, β (95% CI)	p-value	Baseline, β (95% CI)	*p*-value	12-month change, β (95% CI)	*p*-value
*SCHOOL (MEASURED)*												
***Average level (LAeq*, *per 5 dB)***												
Street	‒1.98 (‒6.27, 2.32)	0.367	‒4.83 (‒7.21, ‒2.45)	<0.001	‒3.92 (‒7.74, -0.09)	0.045	‒4.01 (‒5.91, ‒2.10)	<0.001	4.22 (‒1.45, 9.90)	0.145	2.07 (0.37, 3.77)	0.017
Playground	‒0.66 (‒5.81, 4.49)	0.801	‒3.68 (‒6.76, -0.61)	0.019	‒3.62 (‒8.30, 1.06)	0.130	‒4.41 (‒6.89, ‒1.94)	<0.001	5.64 (‒1.04, 12.32)	0.098	1.99 (‒0.20, 4.17)	0.075
Indoor	‒2.65 (‒7.79, 2.48)	0.311	0.14 (‒3.38, 3.66)	0.937	‒1.94 (‒6.52, 2.63)	0.405	‒0.44 (‒3.25, 2.38)	0.762	5.77 (‒0.03, 11.58)	0.051	1.11 (‒1.37, 3.59)	0.381
Individual indoor	0.02 (‒4.17, 4.20)	0.994	‒1.95 (‒5.61, 1.71)	0.296	0.00 (‒3.53, 3.52)	0.999	‒0.80 (‒3.78, 2.19)	0.601	1.28 (‒1.52, 4.07)	0.371	2.41 (0.00, 4.81)	0.050
***Intermittency ratio (per 10%)***												
Street	1.03 (‒1.53, 3.58)	0.431	0.71 (‒1.29, 2.71)	0.486	1.34 (‒1.08, 3.75)	0.278	‒0.66 (‒2.25, 0.94)	0.419	‒2.08 (‒5.38, 1.21)	0.216	0.43 (‒1.00, 1.87)	0.554
Playground	‒0.72 (‒4.55, 3.10)	0.710	‒2.59 (‒5.45, 0.27)	0.075	‒3.07 (‒6.45, 0.31)	0.075	‒3.42 (‒5.71, ‒1.13)	0.003	‒0.31 (‒5.36, 4.74)	0.904	3.76 (1.71, 5.81)	<0.001
Indoor	1.65 (‒1.67, 4.97)	0.330	‒2.27 (‒4.67, 0.13)	0.063	0.15 (‒2.89, 3.19)	0.923	‒2.76 (‒4.66, -0.85)	0.005	‒1.45 (‒5.46, 2.56)	0.479	3.05 (1.34, 4.76)	<0.001
***Number of events (per 50)***												
Street	0.58 (‒3.21, 4.36)	0.765	‒4.38 (‒7.08, ‒1.67)	0.002	‒1.08 (‒4.61, 2.44)	0.547	‒3.99 (‒6.16, -1.82)	<0.001	‒0.25 (‒5.15, 4.65)	0.920	2.13 (0.18, 4.08)	0.032
Playground	‒0.68 (‒4.84, 3.48)	0.749	‒2.68 (‒5.71, 0.35)	0.082	‒3.04 (‒6.85, 0.78)	0.119	‒3.03 (‒5.46, -0.61)	0.014	‒0.77 (‒6.70, 5.16)	0.800	2.22 (0.07, 4.37)	0.043
Indoor	2.04 (‒2.39, 6.46)	0.367	‒2.72 (‒5.45, 0.01)	0.051	‒1.18 (‒5.14, 2.78)	0.559	‒3.22 (‒5.39, ‒1.04)	0.004	‒1.27 (‒6.55, 4.01)	0.636	3.15 (1.20, 5.11)	0.002
*SCHOOL & HOME (MODELLED)*												
***Average level (LAeq*, *per 5 dB)***												
School street, Lday^a^	‒2.98 (‒8.78, 2.82)	0.314	‒6.17 (‒8.84, ‒3.49)	<0.001	‒4.15 (‒9.14, 0.85)	0.104	‒4.91 (‒7.03, ‒2.78)	<0.001	3.42 (‒4.45, 11.29)	0.394	2.81 (0.89, 4.74)	0.004
Home street, Lden^b^	0.00 (‒2.39, 2.40)	0.997	1.35 (‒0.83, 3.53)	0.224	0.75 (‒1.11, 2.61)	0.430	0.40 (‒1.32, 2.12)	0.648	0.72 (‒1.25, 2.68)	0.474	‒0.52 (‒2.06, 1.01)	0.505

^a^*n* = 34.

^b^*n* = 2,346.

Linear mixed models adjusted for age, sex, maternal education, socioeconomical vulnerability index at home, outdoor or indoor TRAP at school or outdoor NO_2_ at home, respectively, for models with the corresponding noise indicators (i.e., outdoors or indoors at school or outdoors at home). Child and school included as nested random effects. The 12-month change models include the term age × corresponding noise indicator to estimate the change; *d*′: detectability, a higher value indicates better working memory; HRT-SE: hit reaction time standard error, a higher value indicates greater inattentiveness; LAeq: A-weighted equivalent noise levels; Lday: LAeq for the daytime (7 AM to 9 PM); Lden: LAeq for the 24 hours with 5 dB and 10 dB penalties for the evening (9 PM to 11 PM) and nighttime (11 PM to 7 AM), respectively; NO_2_: nitrogen dioxide; TRAP: traffic-related air pollution.

#### Associations with average noise levels: School and home

At baseline, i.e., cross-sectionally, we observed some statistically significant associations (S4 Table) between average noise levels at school and the cognitive outcomes, which disappeared in adjusted models (Table 3), except for an association between street average noise levels and complex working memory, β = ‒3.92 points (95% CI: ‒7.74, ‒0.09, *p*-value = 0.045) per 5 dB and a general tendency in the expected direction in the estimated magnitudes for school average noise levels in the street, playground, and indoors in the classroom.

Regarding the 12-month change, both in unadjusted (S4 Table) and adjusted models (see Table 3), school-outdoor average noise levels (both at street level and at the playground) were consistently associated with a slower development of working memory, complex working memory, and with a slower improvement of inattentiveness over 12 months, which reached statistical significance except for the association between playground noise and inattentiveness. For example, a 5-dB increase in street average noise levels was related to a 12-month change of ‒4.83 points (95% CI: ‒7.21, ‒2.45, *p*-value < 0.001) in 2-back detectability, ‒4.01 points (95% CI: ‒5.91, ‒2.10, *p*-value < 0.001) in 3-back detectability, and 2.07 ms (95% CI: 0.37, 3.77, *p*-value = 0.017) in HRT-SE.

Regarding school-indoor average noise levels, individual average noise exposure in classrooms was associated with a slower improvement of inattentiveness in unadjusted models and was borderline significant in adjusted models and of similar magnitude to results with outdoor levels: β = 2.41 ms (95% CI: 0.00, 4.81, *p*-value = 0.050) per 5 dB (adjusted model). In contrast, no association with indoor noise levels was present for working memory and complex working memory (e.g., ‒0.80 points; 95% CI: ‒3.78, 2.19, *p*-value = 0.601, in the 12-month change in 3-back detectability per 5 dB). Indoor average noise levels based on measurements in 1 classroom were not associated with any of the outcomes, although the estimated magnitude was in the expected direction (e.g., 1.11 ms (95% CI: ‒1.37, 3.59, *p*-value = 0.381) in the 12-month change in HRT-SE per 5 dB) (Table 3).

There was no association between home-outdoor average noise levels (Lden) and any of the cognitive outcomes.

#### Associations with noise fluctuation indicators at school

Both school-outdoor and indoor exposure to noise intermittency and to noise events were associated with slower development in the cognitive outcomes in children over 12 months in unadjusted (S4 Table) and adjusted models (Table 3). Some associations were observed at baseline in unadjusted models (S4 Table); however, they disappeared after further adjustment (Table 3).

Regarding intermittency ratio (Table 3), a 10% increment in playground IR was associated with a 12-month change of ‒2.59 points (95% CI: ‒5.45, 0.27, *p*-value = 0.075) in 2-back detectability, ‒3.42 points (95% CI: ‒5.71, ‒1.13, *p*-value = 0.003) in 3-back detectability, and 3.76 ms (95% CI: 1.71, 5.81, *p*-value < 0.001) in HRT-SE, and a 10% increment in indoor IR was associated with a 12-month change of ‒2.27 points (95% CI: ‒4.67, 0.13, *p*-value = 0.063) in 2-back detectability, ‒2.76 points (95% CI: ‒4.66, ‒0.85, *p*-value = 0.005) in 3-back detectability, and 3.05 ms (95% CI: 1.34, 4.76, *p*-value < 0.001) in HRT-SE. Street IR was not associated with any of the outcomes.

Associations were also observed for street, playground, and indoor NE with a slower development in 2-back and 3-back detectability and a slower improvement of inattentiveness over 12 months, which were statistically significant except for playground and indoor NE with 2-back detectability (Table 3). For instance, increments in exposure of 50 noise events at street, playground, and indoor level were associated, respectively, with a 12-month change of ‒4.38 points (95% CI: ‒7.08, ‒1.67, *p*-value = 0.002), ‒2.68 (95% CI: ‒5.71, 0.35, *p*-value = 0.082), and ‒2.72 (95% CI: ‒5.45, 0.01, *p*-value = 0.051) in 2-back detectability, and ‒3.99 points (95% CI: ‒6.16, ‒1.82, *p*-value < 0.001), ‒3.03 (95% CI: ‒5.46, ‒0.61, *p*-value = 0.014), and ‒3.22 (95% CI: ‒5.39, ‒1.04, *p*-value = 0.004) in 3-back detectability, and 2.13 ms (95% CI: 0.18, 4.08, *p*-value = 0.032), 2.22 (95% CI: 0.07, 4.37, *p*-value = 0.043), and 3.15 (95% CI: 1.20, 5.11, *p*-value = 0.002) in HRT-SE.

### Trajectories of working memory and inattentiveness at schools above and below recommended noise levels

**Fig 1:**
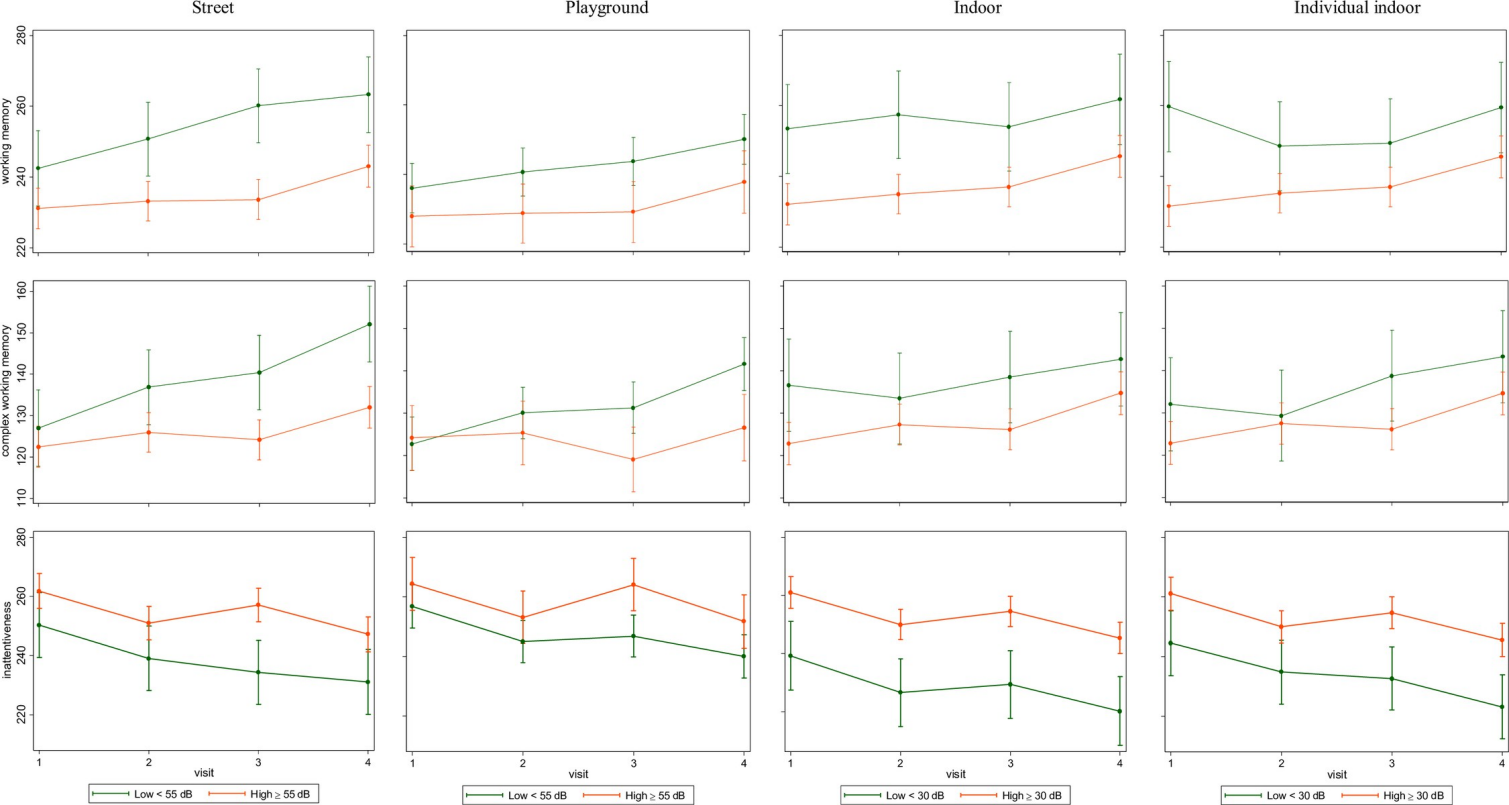
Annual trajectories of working memory, complex working memory, and inattentiveness in children attending schools with low and high average road traffic noise levels (LAeq, dB) outdoors in street and playground or indoors in 1 classroom (indoor level) or in each child’s classroom considering change of room between years (individual indoor level). Y axis: point estimate (beta coefficient), error bars (95% confidence intervals). Predictions for working memory (2-back number stimuli, *d*′), complex working memory (3-back number stimuli, *d*′), and inattentiveness (HRT-SE, ms) adjusted at the means of age, sex, corresponding road traffic noise indicator, age*road traffic noise indicator, maternal education, socioeconomical vulnerability index at home and outdoor or indoor TRAP at school for models including outdoor or indoor noise levels, respectively. Child and school included as nested random effects. *d*′: detectability, a higher value indicates better working memory; HRT-SE: hit reaction time standard error, a higher value indicates greater inattentiveness; LAeq: A-weighted equivalent noise levels; TRAP: traffic-related air pollution.

As shown in Fig 1, children who attended schools with high road traffic noise at street level (≥55 dB) had a slower development of working memory, complex working memory, and a slower improvement of inattentiveness over 12 months than those attending quieter schools in adjusted models. Similar trends with slightly weaker differences between groups were observed for schools exposed to high noise at the playground. Finally, children who attended schools with high road traffic noise in the classroom (≥30 dB) had a slower improvement of inattentiveness over 12 months than those who attended schools with quieter classrooms.

### Sensitivity analyses

When using modelled (Lday) instead of measured average road traffic noise levels at the school street (see bottom of Table 3), we observed the same consistent associations with a slower development of all the cognitive outcomes over 12 months.

Associations between the different road traffic noise indicators at school and the development of cognitive outcomes were robust to additional adjustment for other potential confounders (S5–S7 Tables) and to the inclusion or exclusion of traffic-related air pollution in the adjustment sets. Although in certain adjustment sets the associations of indoor intermittency ratio and indoor number of noise events with working memory gained significance and the association between individual indoor LAeq and inattentiveness lost significance, the estimated magnitudes of effect remained unchanged.

## Discussion

In the current study, exposure to road traffic noise at school, but not at home, was associated with a slower development of working memory and of complex working memory, and with a slower improvement of inattentiveness over 1 year in schoolchildren. Exposure to road traffic noise (at school and home) was not associated with the cognitive outcomes cross-sectionally (i.e., at the baseline visit). Both the school-outdoor average level and fluctuation characteristics of noise were associated with a deceleration in the studied cognitive outcomes. In contrast, in the classroom, the noise fluctuation characteristics were more robustly associated with all cognitive outcomes, whereas average levels were only associated with greater inattentiveness. Finally, children attending schools exposed to outdoor road traffic noise levels ≥55 dB had a slower development of working and complex working memory and greater inattentiveness and those with classroom levels ≥30 dB had greater inattentiveness over 12 months, compared to children exposed to lower outdoor (<55 dB) and indoor (<30 dB) noise levels, respectively.

Associations with working memory (2-back detectability) were generally in the expected direction but only reached statistical significance in relation to street-level indicators (average noise and number of events). This could relate to the fact that the 2-back detectability test was less challenging for children, thus potentially less sensitive to capture changes in development than complex working memory (3-back detectability) for which associations with noise indicators were statistically significant.

As we observed, while individual exposure to the annual average noise level in the classroom was only associated with inattentiveness, exposure to intermittent noise and to a greater number of noise events in the classroom was associated both with greater inattentiveness and slower complex working memory and also marginally with slower working memory development. These findings support the hypothesis that the noise characteristics beyond the average noise level, i.e., its fluctuation, might be more relevant for children’s neurodevelopment in the classroom. They also support the importance of carrying out detailed indoor noise exposure assessment in studies of the cognitive effects of noise, to move closer to the personal exposure inside the classroom. In other words, the peaks of road traffic noise that propagate into the classroom (and their frequency) could be further disruptive for children’s working memory and attention development during concentration at school even when the average noise level in the classroom is lower and may only affect attention. The relevance of the same fluctuation characteristics of noise was previously observed for the effects of transportation noise on cardiovascular outcomes [24]. These results suggest that noise fluctuation should be further investigated and that policy recommendations to protect children’s health may have to consider noise fluctuation in addition to average noise levels. We did not observe associations between exposure to road traffic noise at home and the studied cognitive outcomes, which is in line with the only previous study assessing both school and home exposure [20,21]. This could suggest that exposure to noise at school, rather than at home, may be more detrimental by affecting vulnerable windows of concentration and learning processes [8]. Another complementary explanation may relate to a greater degree of exposure misclassification for noise exposure at home, as we could only assign outdoor average noise levels at the home address, as commonly done in previous studies. Moreover, we could not estimate fluctuation measures, which were relevant indicators associated with cognitive development at school.

We observed very few associations between exposure to road traffic noise at school and the cognitive outcomes at baseline. The baseline results represent the cross-sectional association between exposure to noise and cognitive outcomes during the first visit. The general lack of associations at baseline could partly indicate that the children’s long-term noise exposure years before baseline was not captured with our exposure assessment, given that we did not have historical information about exposure of children in previous schools, although a 98% of children had attended the same school for at least 1 year. Alternatively, the more consistent associations with the 12-month development might respond to the vulnerable window of effects of the studied cognitive functions, which develop significantly during primary school age [14–16]. Finally, inconsistencies in the baseline results could be partly inherent to the cross-sectional nature of the observation, and would be in line with the mixed findings of previous cross-sectional studies [17–23].

### Comparison with previous literature

There is limited evidence for the association between road traffic noise and cognitive development in children, including working memory and attention, which are crucial for learning and school attainment [12,13] and which are actively developing during primary school age [14]. The few studies so far were all cross-sectional and used diverse outcome tests, which precludes direct comparison with the current study. The multicentric and cross-sectional RANCH study, carried out in 2,844 schoolchildren of 9 to 10 years of age in the Netherlands, Spain, and the UK in 2002, observed associations between annual average noise levels of road traffic noise outdoors at schools (daytime LAeq) and cognitive performance, in terms of reading comprehension and episodic memory [19,23]. In line with our study, they observed associations between school-outdoor road traffic noise levels at school, but not at home, and inattention in the 553 children in the Netherlands, based on the Switching Attention Test [20,21]. However, the RANCH project did not find associations with working memory, assessed with a modified version of The Search and Memory Task [19–23], or with sustained attention [19,20,22], assessed with a modified version of the Toulouse–Pieron test [19]. Finally, 2 small studies, one studying exposure to road traffic noise at home in 73 children and another one at 2 schools, found respectively no [17] and suggestive [18] associations with tests of attention. None of the aforementioned studies evaluated exposure to road traffic noise indoors in the classroom or noise fluctuation measures.

### Biological mechanisms

Our results support the general hypothesis that childhood may be a vulnerable period in which external stimuli such as noise could affect the rapid cognitive development that is occurring [7]. There are several suggested pathways on how noise may affect cognitive development in children. One suggested pathway is that noise may affect cognition by impairing attention [8], which is in line with our findings with inattentiveness. It is also suggested that noise could directly impair children’s cognitive abilities, or act indirectly through frustration, learned helplessness, increased arousal, or tuning out, which could impact performance and learning in the long term under repeated noise exposure [8]. The effects of noise on cognition are further supported by animal experiments showing that exposure to environmental noise levels (72 dB, aircraft, up to 4 days) increases oxidative stress in the frontal cortex [11] and subchronic exposure to white noise (4 hours/day, 100 dB, up to 30 days) leads to brain changes in occipital and prefrontal regions [9,10] and in the hippocampus [10], which are relevant for executive functions, learning, and memory. Overall, the suggested pathways and our results support the hypothesis that the effects of noise on cognition, here executive functions, such as working memory, and attention, may be greater during activities that involve concentration or attention and that may affect learning.

### Strengths and limitations

The major strength of this study was the longitudinal design with repeated cognitive evaluations, which allowed to study for the first time the association between exposure to road traffic noise and the development in the cognitive functions of working memory and attention in schoolchildren. We also used validated computerised tests that could capture different dimensions of cognition and executive functions that are developing in primary schoolchildren [34]. Moreover, to our knowledge, this study performed the most extensive and detailed noise exposure assessment of road traffic noise to date, including exposure in different microenvironments at school (at street level, playground, and indoors in the classroom), assessing not only average noise levels but also fluctuation characteristics of noise at school (i.e., intermittency ratio and number of noise events), and exposure to average road traffic noise levels outdoors at home. Furthermore, our road traffic noise measurement protocol yielded representative estimates of the annual average noise levels during the study period, as shown by the high correlation between the annual average noise levels from Barcelona’s Strategic noise map published in 2012 and our school measurements at the street level in 2012 (Spearman’s rank r = 0.86, Pearson’s r = 0.85), the consistent results found with these 2 exposures (modelled and measured) and also the high intraclass correlation (ICC = 0.94) between indoor noise measurements in the 2 campaigns, which were performed 6 months apart.

Among the limitations, our study did not assess past noise exposure. However, all children had attended the same school at least for 6 months and 98% had attended for more than 1 year. In turn, it is unlikely that road traffic noise levels were different during the previous years, as it would require drastic changes in traffic. The lack of association between exposure to noise at home and the cognitive outcomes could partly relate to the greater exposure misclassification expected with the residential assignment of modelled noise levels at the home address. Another limitation is the potential lack of external validity of our results, given that 58% of the participating mothers had a university degree compared to a 50% of women between 25 and 39 years of age in the region [49]. However, maternal education did not seem to determine participation, given that the participation rate was independent of the school-area socioeconomic level (Spearman’s rank correlation = ‒0.09, *p*-value = 0.61). Besides, we could not study children with specific needs, as this would require a larger sample size to have statistical power. Furthermore, residual confounding is always a possibility, although we adjusted for multiple potential confounders and results were robust to additional adjustments in sensitivity analysis. Finally, air pollution could be an important confounder of the studied association, given that it has been associated with impaired cognitive development [26]. In the current study, we adjusted our analyses for TRAP. Moreover, the correlation between TRAPs and the measured noise fluctuation indicators was moderate or low, which further supports that the observed associations between road traffic noise and deceleration in working memory and attention development were independent of air pollution.

### Public health relevance

At least 1 in every 5 Europeans are exposed to high road traffic noise levels (Lden ≥ 55 dB) [1]. In Barcelona, more than half of the population are affected by road traffic noise levels above those recommended by the WHO (Lden ≥ 53 dB) [4,27], and in the current study, more than half of the schools were also exceeding the recommended WHO thresholds both outside and inside classrooms. The current findings suggest that exposure to road traffic noise is associated with a slower development of the cognitive functions of working memory and attention in primary schoolchildren of Barcelona, which are essential for learning and school attainment [12,13]. Given the expected large number of children exposed to road traffic noise at schools, particularly in urban areas, the application of policies to reduce road traffic noise at schools (outside and inside classrooms) could substantially benefit cognitive development, at least working memory and attention, and future health. Furthermore, the current findings add to the previous evidence about the adverse effects of school exposure to aircraft noise on other dimensions of children’s cognition [5,8], and also to our previous findings for the associations of air pollution [26,50,51] with working memory and attention in the same cohort of children. Together, this evidence indicates that efficient interventions to protect the school environment should target transportation and consider not only cleaner air but also quieter school environments.

In conclusion, exposure to road traffic noise at school, but not at home, was associated with slower working memory, complex working memory, and attention development, in primary school children. Associations were observed both for school-outdoor average noise levels and noise fluctuation indicators, although in classrooms, noise fluctuation was more consistently associated with all cognitive outcomes than average noise levels. Finally, slower development of working memory, complex working memory, and attention was observed in children attending schools exposed to outdoor road traffic noise levels above ≥55 dB and to classroom levels ≥30 dB, compared to children exposed to lower outdoor (<55 dB) and indoor (<30 dB) noise levels, respectively. Further longitudinal studies are needed to replicate these findings in different populations and settings, to assess different microenvironments and noise fluctuation metrics and to study other cognitive functions developing over the first years of life.

## Supporting information

S1 STROBE Checklist STROBE Statement—Checklist of items that should be included in reports of cohort. (PDF)

S1 Table Percent or median (interquartile range) of paired school characteristics by low or high school nitrogen dioxide levels. (PDF)

S2 Table Main school- and individual-level characteristics of the study sample by the median of street-outdoor average noise levels at school (LAeq, dB). (PDF)

S3 Table Pearson correlations between noise and traffic-related air pollution levels at schools (*n* = 38) and at individual level (*n* = 2,680 children). (PDF)

S4 Table Estimated unadjusted effect (β) and 95% confidence intervals (95% CI) in cognitive outcomes at baseline and their 12-month change in association to school and home exposure to road traffic noise (*n* = 2,680 children, 9,984 repeats). (PDF)

S5 Table Unadjusted and additionally adjusted models for the association between school exposure to average noise levels (LAeq, per 5 dB change) and 12-month change of working memory, complex working memory, and inattentiveness. (*n* = 2,680 children, 9,984 repeats).
(PDF)

S6 Table Unadjusted and additionally adjusted models for the association between school exposure to noise intermittency ratio (IR, per 10% change) and 12-month change of working memory, complex working memory, and inattentiveness (*n* = 2,680 children, 9,984 repeats). (PDF)

S7 Table Unadjusted and additionally adjusted models for the association between school exposure to number of noise events (NE, per 50 events) and 12-month change of working memory, complex working memory, and inattentiveness (*n* = 2,680 children, 9,984 repeats). (PDF)

S1 Text Estimation of the individual indoor noise levels in the classroom. (PDF)

## Abbreviations

ANT: Attentional Network Test
BREATHE: Brain Development and Air Pollution Ultrafine Particles in School Children
*d*′: detectability
EC: elemental carbon
HRT-SE: hit reaction time standard error
IR: Intermittency Ratio
IQR: interquartile range
LAeq: A-weighted equivalent noise levels
Lday: LAeq for the daytime (7 AM to 9 PM)
Lden: LAeq for the 24 hours with 5 dB and 10 dB penalties for the evening (9 PM to 11 PM) and nighttime (11 PM to 7 AM), respectively
NE: noise event
NO_2_: nitrogen dioxide
PISA: Programme for International Student Assessment
RANCH: road traffic and aircraft noise exposure and children’s cognition and health
SDQ: Strengths and Difficulties Questionnaire
TRAP: traffic-related air pollution
