# Supplementary material for: Exposure to road traffic noise and cognitive development in schoolchildren in Barcelona, Spain: A population-based cohort study
Source: PLoS Med. 2022 Jun 2;19(6):e1004001. doi: 10.1371/journal.pmed.1004001 (PMC9162347; doi:10.1371/journal.pmed.1004001)
Supplement: S1 STROBE Checklist — (PDF) [file pmed.1004001.s001.pdf]

S1 STROBE Checklist

STROBE Statement—Checklist of items that should be included in reports of cohort studies

	Item No	Recommendation	Section and paragraph number
**Title and abstract**	1	(*a*) Indicate the study's design with a commonly used term in the title or the abstract	Title, p1<br>Abstract, p2
		(*b*) Provide in the abstract an informative and balanced summary of what was done and what was found	Abstract, p2-3
**Introduction**			
Background/rationale	2	Explain the scientific background and rationale for the investigation being reported	Introduction, p1-5
Objectives	3	State specific objectives, including any prespecified hypotheses	Introduction, p6
**Methods**			
Study design	4	Present key elements of study design early in the paper	Study population and design, p1
Setting	5	Describe the setting, locations, and relevant dates, including periods of recruitment, exposure, follow-up, and data collection	Study population and design, p1<br>Noise exposure assessment, p1<br>Outcomes, p1
Participants	6	(*a*) Give the eligibility criteria, and the sources and methods of selection of participants. Describe methods of follow-up	Study population and design, p1.
		(*b*) For matched studies, give matching criteria and number of exposed and unexposed	Study population and design, p1
Variables	7	Clearly define all outcomes, exposures, predictors, potential confounders, and effect modifiers. Give diagnostic criteria, if applicable	Methods, p4-18
Data sources/ measurement	8*	For each variable of interest, give sources of data and details of methods of assessment (measurement). Describe comparability of assessment methods if there is more than one group	Methods, p4-18
Bias	9	Describe any efforts to address potential sources of bias	Methods, p21-24
Study size	10	Explain how the study size was arrived at	Methods, p21
Quantitative variables	11	Explain how quantitative variables were handled in the analyses. If applicable, describe which groupings were chosen and why	Methods, p21
Statistical methods	12	(*a*) Describe all statistical methods, including those used to control for confounding	Methods, p21-24
		(*b*) Describe any methods used to examine subgroups and interactions	Methods, p22
		(*c*) Explain how missing data were addressed	Methods, p21
		(*d*) If applicable, explain how loss to follow-up was addressed	Methods, p21
		(*e*) Describe any sensitivity analyses	Methods, p23

**Results**			
Participants	13*	(a) Report numbers of individuals at each stage of study—eg numbers potentially eligible, examined for eligibility, confirmed eligible, included in the study, completing follow-up, and analysed	Methods, p1, p21
		(b) Give reasons for non-participation at each stage	Methods p1, p9, p21
		(c) Consider use of a flow diagram	NA
Descriptive data	14*	(a) Give characteristics of study participants (eg demographic, clinical, social) and information on exposures and potential confounders	Results, p1-2
		(b) Indicate number of participants with missing data for each variable of interest	Results, Table 2, Table 3
		(c) Summarise follow-up time (eg, average and total amount)	Abstract, p2 Outcomes, p1, Results, p1
Outcome data	15*	Report numbers of outcome events or summary measures over time	Results, p1
Main results	16	(*a*) Give unadjusted estimates and, if applicable, confounder-adjusted estimates and their precision (eg, 95% confidence interval). Make clear which confounders were adjusted for and why they were included	Results, p4-11
		(*b*) Report category boundaries when continuous variables were categorized	Results, p12
		(*c*) If relevant, consider translating estimates of relative risk into absolute risk for a meaningful time period	NA
Other analyses	17	Report other analyses done—eg analyses of subgroups and interactions, and sensitivity analyses	Results, p14-15
**Discussion**			
Key results	18	Summarise key results with reference to study objectives	Discussion, p1
Limitations	19	Discuss limitations of the study, taking into account sources of potential bias or imprecision. Discuss both direction and magnitude of any potential bias	Discussion, p8
Interpretation	20	Give a cautious overall interpretation of results considering objectives, limitations, multiplicity of analyses, results from similar studies, and other relevant evidence	Discussion, p1-10
Generalisability	21	Discuss the generalisability (external validity) of the study results	Abstract, p3 Discussion, p8
**Other information**			
Funding	22	Give the source of funding and the role of the funders for the present study and, if applicable, for the original study on which the present article is based	Funding statement, p1

*Give information separately for exposed and unexposed groups.

Note: An Explanation and Elaboration article discusses each checklist item and gives methodological background and published examples of transparent reporting. The STROBE checklist is best used in conjunction with this article (freely available on the Web sites of PLoS Medicine at http://www.plosmedicine.org/, Annals of Internal Medicine at http://www.annals.org/, and Epidemiology at http://www.epidem.com/). Information on the STROBE Initiative is available at http://www.strobe-statement.org.
