# Supplementary material for: Exposure to road traffic noise and cognitive development in schoolchildren in Barcelona, Spain: A population-based cohort study
Source: PLoS Med. 2022 Jun 2;19(6):e1004001. doi: 10.1371/journal.pmed.1004001 (PMC9162347; doi:10.1371/journal.pmed.1004001)
Supplement: S1 Table — (PDF) [file pmed.1004001.s002.pdf]

S1 Table. Percent or median (interquartile range) of paired school characteristics by low or high school nitrogen dioxide levels.

	Low NO_2 (n=19)	High NO_2 (n=19)	p-value[a]
Modeled school NO_2 ($\mu g/m^3$)	30.49 (25.12)	59.41 (6.00)	0.0001
School SES vulnerability index (n)	0.55 (0.48)	0.42 (0.39)	0.2083
Type of school, public (%)	57.9	42.1	0.3300

[a] Kruskal Wallis test for continuous variables or Chi^2 test for categorical variables. NO_2: Modeled school nitrogen dioxide levels. Low NO_2 ≤ 51 $\mu g/m^3$ / High NO_2 > 51 $\mu g/m^3$. SES: socio-economic status.
