# Supplementary material for: Exposure to road traffic noise and cognitive development in schoolchildren in Barcelona, Spain: A population-based cohort study
Source: PLoS Med. 2022 Jun 2;19(6):e1004001. doi: 10.1371/journal.pmed.1004001 (PMC9162347; doi:10.1371/journal.pmed.1004001)
Supplement: S2 Table — (PDF) [file pmed.1004001.s003.pdf]

S2 Table. Main school- and individual-level characteristics of the study sample by the median of street-outdoor average noise levels at school (LAeq, dB).

**Variables**	**LAeq < p50[a]**<br><br>**Median (IQR) or %**	**LAeq ≥ p50[a]**<br><br>**Median (IQR) or %**	**p-value[b]**
**School-level variables (n=38)**			
Type of school, public	55%	44.4%	0.516
School socioeconomic vulnerability index (n)	0.5 (0.5)	0.4 (0.3)	0.076
School education quality (PISA 2012, n)	5.0 (3.0)	3.0 (2.0)	< 0.001
**Individual-level variables (n=2680)**			
2-back number stimuli (d')[c]	220.9 (261.1)	205.1 (231.9)	0.127
3-back number stimuli (d')[d]	111.8 (131.0)	111.8 (125.0)	0.087
ANT (HRT-SE)[e]	262.3 (134.5)	271.9 (142.1)	0.012
Age (years)	8.5 (1.5)	8.5 (1.4)	0.194
Girls	49.6%	50.3%	0.731
Maternal education, university	62.9%	54.1%	< 0.001
Paternal education, university	57.1%	48.7%	< 0.001
Foreign origin (non-Spanish)	10.5%	19.6%	< 0.001
Marital status, married	86.7%	83.3%	0.015
Overweight, Yes	25.7%	29.7%	0.026
Computer games weekend, > 1h	68.5%	72.5%	0.032
Siblings, Yes	82.1%	75.8%	< 0.001
Adopted child, Yes	4.3%	3.5%	0.276
Smoking during pregnancy, Yes	10.9%	9.4%	0.197
Birth ≥ 37 weeks	92.2%	92%	0.882
Birth weight ≥ 2.5 kg	90.2%	89.8%	0.736
Breastfeeding, Yes	82.5%	81.4%	0.478
Behavioural problems (SDQ)	7.0 (7.0)	8.0 (7.0)	< 0.001
Home socioeconomic vulnerability index (n)	0.4 (0.4)	0.5 (0.3)	< 0.001

[a]Median Street LAeq, dB = 63.5 dB. LAeq: A-weighted equivalent noise levels. [b]Kruskal Wallis test for continuous variables or Chi^2 test for categorical variables. PISA: Programme for International Student Assessment. [c]Working memory, [d]Complex working memory. d': detectability, a higher value indicates better working memory, [e]Inattentiveness: Hit Reaction Time Standard Error (HRT-SE, ms) of the Attention Network Task, a higher value indicates greater inattentiveness. SDQ: Strengths and Difficulties Questionnaire.
