# Supplementary material for: Exposure to road traffic noise and cognitive development in schoolchildren in Barcelona, Spain: A population-based cohort study
Source: PLoS Med. 2022 Jun 2;19(6):e1004001. doi: 10.1371/journal.pmed.1004001 (PMC9162347; doi:10.1371/journal.pmed.1004001)
Supplement: S3 Table — (PDF) [file pmed.1004001.s004.pdf]

S3 Table. Pearson correlations between noise and traffic-related air pollution levels at schools (n=38), and at individual-level (n=2680 children)

		SCHOOL													HOME	
	(r, p-value)	LAeq, street	LAeq, playgr.	LAeq, in	LAeq, in (indiv.)	IR, street	IR, playgr.	IR, in	NE, street	NE, playgr.	NE, in	TRAPs, in	TRAPs, out	Lday, street	NO_2, out	Lden, out
SCHOOL	LAeq, street (dB)	1.00, <0.001														
	LAeq, playground (dB)	0.74, <0.001	1.00, <0.001													
	LAeq, indoor (dB)	0.34, 0.038	0.47, 0.004	1.00, <0.001												
	LAeq, indoor (individual) (dB)	0.36, <0.001	0.44, <0.001	0.63, <0.001	1.00, <0.001											
	IR, street (%)	-0.19, 0.251	-0.34, 0.044	-0.45, 0.005	-0.10, 0.547	1.00, <0.001										
	IR, playground (%)	0.38, 0.023	0.32, 0.059	0.05, 0.758	0.03, 0.870	0.15, 0.377	1.00, <0.001									
	IR, indoor (%)	0.26, 0.110	0.14, 0.426	0.14, 0.405	0.37, 0.022	0.29, 0.078	0.40, 0.017	1.00, <0.001								
	NE, street (number)	0.64, <0.001	0.37, 0.029	0.00, 0.988	0.33, 0.042	0.29, 0.074	0.33, 0.050	0.32, 0.053	1.00, <0.001							
	NE, playground (number)	0.51, 0.002	0.49, 0.002	0.24, 0.161	0.11, 0.536	0.02, 0.919	0.82, <0.001	0.30, 0.081	0.50, 0.002	1.00, <0.001						
	NE, indoor (number)	0.43, 0.007	0.30, 0.076	0.37, 0.024	0.45, 0.004	0.11, 0.500	0.26, 0.123	0.83, <0.001	0.43, 0.007	0.31, 0.067	1.00, <0.001					
	TRAP, indoor	0.67, <0.001	0.58, <0.001	0.54, <0.001	0.51, 0.001	-0.22, 0.191	0.11, 0.529	0.27, 0.100	0.36, 0.027	0.22, 0.207	0.54, <0.001	1.00, <0.001				
	TRAP, outdoor	0.70, <0.001	0.68, <0.001	0.46, 0.004	0.48, 0.003	-0.15, 0.384	0.28, 0.094	0.27, 0.107	0.40, 0.014	0.41, 0.012	0.44, 0.005	0.79, <0.001	1.00, <0.001			
	Lday, street [a]	0.85, <0.001	0.70, <0.001	0.37, 0.033	0.38, 0.029	-0.43, 0.012	0.36, 0.043	0.26, 0.135	0.50, 0.003	0.54, 0.001	0.39, 0.023	0.59, <0.001	0.66, <0.001	1.00, <0.001		
HOME	NO_2, outdoor ($\mu g/m^3$)	0.30, <0.001	0.23, <0.001	0.19, <0.001	0.18, <0.001	-0.09, <0.001	0.19, <0.001	0.01, 0.513	0.15, <0.001	0.18, <0.001	0.10, <0.001	0.29, <0.001	0.28, <0.001	0.21, <0.001	1.00, <0.001	
	Lden, outdoor (dB) [b]	-0.01, 0.581	-0.01, 0.629	0.03, 0.131	0.08, <0.001	-0.01, 0.505	-0.03, 0.165	0.04, 0.052	-0.03, 0.093	-0.02, 0.350	0.02, 0.328	0, 0.854	0.01, 0.594	0.02, 0.287	0.33, <0.001	1.00, <0.001

[a] n=34; [b] n=2346. LAeq: Measured A-weighted equivalent noise level, IR: Measured noise intermittency Ratio, NE: Measured Number of noise Events, TRAP: Measured Traffic-related air pollution (NO_2 and EC), NO_2: Modelled nitrogen dioxide, Lday: LAeq for the day-time (7 a.m.-9 p.m.), Lden: LAeq for the 24h (Lden) with 5 dB and 10 dB penalties for the evening (9 p.m. to 11 p.m.) and night-time (11 p.m. to 7 a.m.), respectively.

Very light blue: r = | 0 to 0.40 |, Medium blue: r = | 0.41 to 0.70 |, darkest blue: r = | 0.71 to 1.00|.
