# Supplementary material for: Exposure to road traffic noise and cognitive development in schoolchildren in Barcelona, Spain: A population-based cohort study
Source: PLoS Med. 2022 Jun 2;19(6):e1004001. doi: 10.1371/journal.pmed.1004001 (PMC9162347; doi:10.1371/journal.pmed.1004001)
Supplement: S4 Table — (PDF) [file pmed.1004001.s005.pdf]

S4 Table. Estimated unadjusted effect (β) and 95% confidence intervals (95%CI) in cognitive outcomes at baseline and their 12-month change in association to school and home exposure to road traffic noise (n = 2680 children, 9984 repeats).

Road traffic noise indicators	Working memory (2-back numbers, d')				Complex working memory (3-back numbers, d')				Inattentiveness (Attention Network task, HRT -SE[ms])			
	Baseline β (95% CI)	p-value	12-mo change β (95% CI)	p-value	Baseline β (95% CI)	p-value	12-mo change β (95% CI)	p-value	Baseline β (95% CI)	p-value	12-mo change β (95% CI)	p-value
SCHOOL (MEASURED)												
Average level (LAeq, per 5dB)												
Street	-6.68 (-11.13, -2.22)	0.003	-4.74 (-7.18, -2.30)	<0.001	-4.70 (-8.11, -1.28)	0.007	-3.95 (-5.89, -2.02)	<0.001	6.08 (1.08, 11.08)	0.017	2.22 (0.49, 3.95)	0.012
Playground	-7.60 (-13.59, -1.61)	0.013	-3.44 (-6.61, -0.26)	0.034	-5.37 (-9.88, -0.85)	0.020	-4.31 (-6.84, -1.78)	<0.001	7.70 (1.41, 13.99)	0.016	2.10 (-0.13, 4.33)	0.064
Indoor	-7.74 (-13.93, -1.55)	0.014	-0.21 (-3.82, 3.40)	0.910	-4.07 (-8.90, 0.76)	0.099	-0.63 (-3.50, 2.23)	0.664	9.79 (3.65, 15.94)	0.002	1.40 (-1.13, 3.92)	0.278
Individual indoor	-1.28 (-5.72, 3.16)	0.572	-2.02 (-5.83, 1.78)	0.298	-0.81 (-4.37, 2.75)	0.655	-0.82 (-3.87, 2.24)	0.600	1.74 (-1.08, 4.56)	0.226	2.57 (0.13, 5.00)	0.039
Intermittency ratio (per 10%)												
Street	1.47 (-2.68, 5.61)	0.488	0.73 (-1.32, 2.78)	0.486	1.42 (-1.72, 4.56)	0.375	-0.63 (-2.25, 0.99)	0.446	-2.57 (-6.84, 1.71)	0.240	0.28 (-1.18, 1.74)	0.704
Playground	-6.58 (-12.12, -1.04)	0.020	-2.29 (-5.22, 0.64)	0.126	-5.94 (-9.83, -2.06)	0.003	-3.34 (-5.66, -1.02)	0.005	3.87 (-2.29, 10.02)	0.218	3.76 (1.67, 5.84)	<0.001
Indoor	-0.86 (-5.82, 4.09)	0.733	-2.19 (-4.65, 0.27)	0.080	-0.90 (-4.61, 2.82)	0.637	-2.76 (-4.70, -0.82)	0.005	0.18 (-5.06, 5.41)	0.947	3.09 (1.35, 4.83)	<0.001
Number of events (per 50)												
Street	-3.56 (-8.96, 1.83)	0.195	-4.27 (-7.06, -1.49)	0.003	-3.15 (-7.21, 0.91)	0.128	-3.93 (-6.14, -1.73)	<0.001	2.49 (-3.27, 8.24)	0.398	2.24 (0.25, 4.23)	0.027
Playground	-7.35 (-13.54, -1.17)	0.020	-2.28 (-5.37, 0.81)	0.147	-6.33 (-10.70, -1.97)	0.004	-2.91 (-5.36, -0.46)	0.020	4.30 (-2.76, 11.36)	0.233	2.31 (0.12, 4.49)	0.038
Indoor	-1.85 (-7.46, 3.77)	0.519	-2.67 (-5.48, 0.14)	0.062	-1.87 (-6.07, 2.32)	0.381	-3.21 (-5.43, -1.00)	0.005	1.96 (-3.97, 7.89)	0.517	3.32 (1.32, 5.31)	0.001
SCHOOL & HOME (MODELLED)												
Average level (LAeq, per 5dB)												
School street, Lday[a]	-5.14 (-10.31, 0.03)	0.051	-6.45 (-9.22, -3.67)	<0.001	-3.81 (-7.72, 0.10)	0.056	-4.99 (-7.18, -2.80)	<0.001	4.73 (-0.92, 10.39)	0.101	3.08 (1.12, 5.04)	0.002
Home street, Lden[b]	0.62 (-1.67, 2.91)	0.597	1.77 (-0.43, 3.97)	0.115	0.71 (-1.06, 2.48)	0.431	0.67 (-1.07, 2.40)	0.450	0.22 (-1.67, 2.10)	0.821	-0.82 (-2.37, 0.74)	0.303

Unadjusted linear mixed models include age, corresponding noise indicator and age × noise indicator to estimate the change. Child and school included as nested random effects. d': detectability: a higher value indicates better working memory, HRT-SE: Hit Reaction Time Standard Error: a higher value indicates greater inattentiveness. LAeq: A-weighted equivalent noise levels, Lday: LAeq for the day-time (7 a.m.-9 p.m.), Lden: LAeq for the 24h (Lden) with 5 dB and 10 dB penalties for the evening (9 p.m. to 11 p.m.) and night-time (11 p.m. to 7 a.m.), respectively. [a] n=34; [b] n=2346.
