# Supplementary material for: Exposure to road traffic noise and cognitive development in schoolchildren in Barcelona, Spain: A population-based cohort study
Source: PLoS Med. 2022 Jun 2;19(6):e1004001. doi: 10.1371/journal.pmed.1004001 (PMC9162347; doi:10.1371/journal.pmed.1004001)
Supplement: S5 Table — (n = 2,680 children, 9,984 repeats). (PDF) [file pmed.1004001.s006.pdf]

S5 Table. Unadjusted and additionally adjusted models for the association between school exposure to average noise levels (LAeq, per 5 dB change) and 12-month change of working memory, complex working memory and inattentiveness. (n = 2680 children, 9984 repeats).

**Working memory (2-back number stimuli (detectability: d'))** [a]								
	LAeq, street		LAeq, playground		LAeq, indoor		LAeq, individual indoor	
Model	β (95%CI)	p-value	β (95%CI)	p-value	β (95%CI)	p-value	β (95%CI)	p-value
M0	-4.74 (-7.18, -2.30)	<0.001	-3.44 (-6.61, -0.26)	0.034	-0.21 (-3.82, 3.40)	0.910	-2.02 (-5.83, 1.78)	0.298
M1	-4.81 (-7.19, -2.43)	<0.001	-3.67 (-6.75, -0.58)	0.020	0.22 (-3.30, 3.74)	0.904	-1.82 (-5.48, 1.85)	0.332
M2	-4.83 (-7.21, -2.45)	<0.001	-3.68 (-6.76, -0.61)	0.019	0.14 (-3.38, 3.66)	0.937	-1.95 (-5.61, 1.71)	0.296
M3	-4.83 (-7.22, -2.44)	<0.001	-3.67 (-6.75, -0.59)	0.020	0.17 (-3.36, 3.70)	0.924	-1.93 (-5.61, 1.75)	0.304
M4	-4.84 (-7.25, -2.42)	<0.001	-3.70 (-6.83, -0.58)	0.020	-0.19 (-3.78, 3.40)	0.918	-2.22 (-5.98, 1.55)	0.249
M5	-4.85 (-7.24, -2.46)	<0.001	-3.93 (-7.00, -0.85)	0.012	-0.16 (-3.69, 3.37)	0.929	-2.21 (-5.90, 1.49)	0.241
M6	-4.97 (-7.37, -2.56)	<0.001	-3.92 (-7.04, -0.80)	0.014	-0.11 (-3.69, 3.47)	0.951	-1.76 (-5.50, 1.98)	0.357
M7	-4.77 (-7.20, -2.35)	<0.001	-3.64 (-6.77, -0.51)	0.023	-0.67 (-4.25, 2.90)	0.713	-2.61 (-6.32, 1.10)	0.168
M8	-4.86 (-7.30, -2.43)	<0.001	-3.99 (-7.15, -0.84)	0.013	-0.57 (-4.17, 3.03)	0.756	-2.50 (-6.26, 1.26)	0.192
M9	-4.79 (-7.19, -2.40)	<0.001	-3.66 (-6.75, -0.57)	0.020	0.28 (-3.27, 3.82)	0.879	-1.91 (-5.62, 1.81)	0.314
M10	-4.88 (-7.28, -2.49)	<0.001	-3.70 (-6.79, -0.61)	0.019	0.21 (-3.33, 3.76)	0.906	-1.61 (-5.32, 2.11)	0.396
M11	-4.75 (-7.17, -2.33)	<0.001	-3.74 (-6.87, -0.61)	0.019	0.17 (-3.43, 3.77)	0.927	-1.57 (-5.34, 2.19)	0.413
M12	-4.91 (-7.34, -2.48)	<0.001	-3.73 (-6.87, -0.58)	0.020	0.39 (-3.23, 4.02)	0.831	-1.76 (-5.55, 2.03)	0.363
M13	-4.99 (-7.41, -2.57)	<0.001	-3.55 (-6.70, -0.40)	0.027	0.27 (-3.35, 3.89)	0.882	-1.85 (-5.64, 1.93)	0.337
M14	-4.76 (-7.15, -2.38)	<0.001	-3.88 (-6.97, -0.79)	0.014	0.10 (-3.45, 3.66)	0.954	-1.75 (-5.46, 1.97)	0.357
M15	-4.95 (-7.33, -2.57)	<0.001	-3.66 (-6.72, -0.59)	0.019	-0.11 (-3.61, 3.40)	0.953	-1.81 (-5.45, 1.84)	0.331
M16	-4.91 (-7.30, -2.53)	<0.001	-4.00 (-7.11, -0.89)	0.012	0.10 (-3.43, 3.64)	0.954	-2.02 (-5.73, 1.70)	0.287
M17	-4.63 (-7.00, -2.26)	<0.001	-3.66 (-6.71, -0.62)	0.018	0.16 (-3.34, 3.66)	0.930	-1.82 (-5.50, 1.85)	0.330
M18	-4.83 (-7.21, -2.45)	<0.001	-3.68 (-6.76, -0.61)	0.019	0.14 (-3.38, 3.66)	0.937	-1.95 (-5.61, 1.71)	0.296

S5 Table (Continued)

Complex working memory (3-back number stimuli (detectability: d')) [a]								
	LAeq, street		LAeq, playground		LAeq, indoor		LAeq, individual indoor	
Model	β (95%CI)	p-value	β (95%CI)	p-value	β (95%CI)	p-value	β (95%CI)	p-value
M0	-3.95 (-5.89, -2.02)	<0.001	-4.31 (-6.84, -1.78)	<0.001	-0.63 (-3.50, 2.23)	0.664	-0.82 (-3.87, 2.24)	0.600
M1	-4.02 (-5.92, -2.11)	<0.001	-4.41 (-6.89, -1.93)	<0.001	-0.45 (-3.27, 2.37)	0.755	-0.78 (-3.76, 2.20)	0.607
M2	-4.01 (-5.91, -2.10)	<0.001	-4.41 (-6.89, -1.94)	<0.001	-0.44 (-3.25, 2.38)	0.762	-0.80 (-3.78, 2.19)	0.601
M3	-4.00 (-5.91, -2.09)	<0.001	-4.41 (-6.89, -1.93)	<0.001	-0.38 (-3.20, 2.45)	0.793	-0.71 (-3.71, 2.28)	0.642
M4	-3.84 (-5.77, -1.91)	<0.001	-4.31 (-6.82, -1.80)	<0.001	-0.13 (-3.00, 2.74)	0.932	-0.41 (-3.47, 2.64)	0.791
M5	-3.87 (-5.78, -1.96)	<0.001	-4.31 (-6.79, -1.83)	<0.001	-0.53 (-3.36, 2.30)	0.714	-0.62 (-3.62, 2.39)	0.686
M6	-3.89 (-5.80, -1.97)	<0.001	-4.27 (-6.77, -1.77)	<0.001	-0.24 (-3.10, 2.63)	0.871	-0.50 (-3.53, 2.53)	0.746
M7	-4.27 (-6.21, -2.32)	<0.001	-4.91 (-7.44, -2.38)	<0.001	-1.31 (-4.19, 1.56)	0.370	-1.54 (-4.57, 1.49)	0.318
M8	-4.29 (-6.24, -2.35)	<0.001	-4.80 (-7.35, -2.25)	<0.001	-0.72 (-3.61, 2.17)	0.625	-1.15 (-4.22, 1.91)	0.461
M9	-3.87 (-5.78, -1.96)	<0.001	-4.27 (-6.75, -1.78)	<0.001	-0.17 (-3.01, 2.66)	0.905	-0.43 (-3.45, 2.58)	0.779
M10	-3.87 (-5.79, -1.96)	<0.001	-4.18 (-6.67, -1.69)	<0.001	-0.14 (-2.97, 2.70)	0.925	-0.21 (-3.23, 2.81)	0.892
M11	-3.73 (-5.66, -1.79)	<0.001	-4.16 (-6.68, -1.63)	0.001	-0.11 (-3.00, 2.77)	0.939	-0.11 (-3.18, 2.96)	0.942
M12	-3.65 (-5.58, -1.71)	<0.001	-4.08 (-6.60, -1.55)	0.002	-0.19 (-3.08, 2.71)	0.900	-0.19 (-3.27, 2.89)	0.903
M13	-3.72 (-5.66, -1.78)	<0.001	-4.40 (-6.94, -1.87)	<0.001	-0.42 (-3.32, 2.49)	0.779	-0.17 (-3.26, 2.92)	0.915
M14	-3.86 (-5.77, -1.95)	<0.001	-4.34 (-6.83, -1.84)	<0.001	-0.41 (-3.26, 2.43)	0.775	-0.59 (-3.63, 2.44)	0.702
M15	-4.11 (-6.02, -2.21)	<0.001	-4.40 (-6.87, -1.92)	<0.001	-0.75 (-3.56, 2.05)	0.600	-0.66 (-3.63, 2.30)	0.661
M16	-4.11 (-6.01, -2.21)	<0.001	-4.81 (-7.31, -2.32)	<0.001	-0.57 (-3.40, 2.25)	0.691	-1.15 (-4.15, 1.86)	0.455
M17	-3.77 (-5.67, -1.87)	<0.001	-4.21 (-6.68, -1.74)	<0.001	-0.37 (-3.19, 2.44)	0.796	-0.65 (-3.65, 2.34)	0.668
M18	-4.01 (-5.91, -2.10)	<0.001	-4.41 (-6.89, -1.94)	<0.001	-0.44 (-3.25, 2.38)	0.762	-0.80 (-3.78, 2.19)	0.601

S5 Table (Continued)

	Inattentiveness (Attention Network Test, Hit reaction time standard error, HRT-SE, ms) [b]							
	LAeq, street		LAeq, playground		LAeq, indoor		LAeq, individual indoor	
Model	β (95%CI)	p-value	β (95%CI)	p-value	β (95%CI)	p-value	β (95%CI)	p-value
M0	2.22 (0.49, 3.95)	0.012	2.10 (-0.13, 4.33)	0.064	1.40 (-1.13, 3.92)	0.278	2.57 (0.13, 5.00)	0.039
M1	2.08 (0.37, 3.78)	0.017	1.99 (-0.20, 4.18)	0.075	1.08 (-1.40, 3.56)	0.393	2.36 (-0.05, 4.76)	0.055
M2	2.07 (0.37, 3.77)	0.017	1.99 (-0.20, 4.17)	0.075	1.11 (-1.37, 3.59)	0.381	2.41 (0.00, 4.81)	0.050
M3	2.04 (0.34, 3.75)	0.019	1.95 (-0.24, 4.14)	0.081	1.05 (-1.43, 3.54)	0.406	2.35 (-0.05, 4.76)	0.055
M4	1.94 (0.22, 3.65)	0.027	1.82 (-0.39, 4.03)	0.107	0.86 (-1.66, 3.38)	0.502	2.08 (-0.37, 4.52)	0.096
M5	1.97 (0.25, 3.68)	0.024	1.98 (-0.22, 4.18)	0.078	1.05 (-1.45, 3.55)	0.411	2.14 (-0.29, 4.57)	0.084
M6	2.03 (0.32, 3.75)	0.020	2.03 (-0.18, 4.23)	0.072	1.07 (-1.45, 3.59)	0.404	2.17 (-0.27, 4.61)	0.081
M7	1.98 (0.25, 3.72)	0.025	1.68 (-0.55, 3.91)	0.141	1.08 (-1.45, 3.60)	0.403	2.41 (-0.02, 4.85)	0.052
M8	2.20 (0.46, 3.94)	0.013	1.85 (-0.40, 4.10)	0.106	1.08 (-1.47, 3.62)	0.407	1.85 (-0.62, 4.32)	0.143
M9	1.98 (0.27, 3.69)	0.023	1.86 (-0.34, 4.06)	0.098	0.93 (-1.57, 3.44)	0.464	2.08 (-0.35, 4.51)	0.094
M10	2.00 (0.28, 3.72)	0.022	1.89 (-0.32, 4.09)	0.094	0.82 (-1.69, 3.33)	0.520	2.06 (-0.37, 4.50)	0.097
M11	2.10 (0.38, 3.83)	0.017	2.04 (-0.18, 4.26)	0.072	1.29 (-1.25, 3.82)	0.320	2.25 (-0.21, 4.72)	0.073
M12	2.05 (0.31, 3.78)	0.021	1.97 (-0.27, 4.20)	0.085	1.50 (-1.06, 4.05)	0.250	2.26 (-0.22, 4.74)	0.074
M13	1.99 (0.25, 3.72)	0.025	1.79 (-0.44, 4.03)	0.116	1.50 (-1.05, 4.06)	0.248	2.01 (-0.47, 4.48)	0.112
M14	1.95 (0.23, 3.66)	0.026	1.98 (-0.23, 4.19)	0.080	1.38 (-1.13, 3.90)	0.282	2.23 (-0.22, 4.68)	0.075
M15	2.09 (0.38, 3.79)	0.016	1.98 (-0.21, 4.17)	0.076	1.17 (-1.31, 3.65)	0.355	2.37 (-0.04, 4.77)	0.054
M16	2.12 (0.42, 3.83)	0.014	2.62 (0.41, 4.82)	0.020	0.82 (-1.67, 3.31)	0.519	3.27 (0.85, 5.70)	0.008
M17	1.77 (0.08, 3.46)	0.040	1.67 (-0.51, 3.84)	0.133	0.98 (-1.49, 3.45)	0.438	2.12 (-0.30, 4.53)	0.085
M18	2.06 (0.36, 3.76)	0.018	1.96 (-0.23, 4.15)	0.079	1.13 (-1.36, 3.61)	0.374	2.47 (0.07, 4.86)	0.044

[a] A higher value in the test indicates better working memory; [b] a higher value in the test indicates greater inattentiveness. M0: Linear mixed models for the unadjusted change (i.e. adjusted for age, corresponding noise indicator × age, with child and school as nested random effects). M1 (Main adjustment set without TRAPs): M0 further adjusted for age, sex, maternal education, socio-economical vulnerability index at home. M2 (Main model): M1 further adjusted for outdoor or indoor traffic-related air pollution (TRAPs) at school, respectively, for models with the corresponding outdoor or indoor noise indicators. M3 to M17 correspond to M2 further adjusted for: M3: type of school, M4: Paternal education, M5: Foreign origin, M6: Marital status, M7: Overweight, M8: Computer games in the weekend, M9: Siblings, M10: Adoption, M11: Smoking during pregnancy, M12: Preterm birth, M13: Birth weight, M14: Breastfeeding, M15: Socio-economical vulnerability index at school, M16: School education quality, M17: Behavioural problems, M18: Paired school by design as nested random effect.
