# Supplementary material for: Exposure to road traffic noise and cognitive development in schoolchildren in Barcelona, Spain: A population-based cohort study
Source: PLoS Med. 2022 Jun 2;19(6):e1004001. doi: 10.1371/journal.pmed.1004001 (PMC9162347; doi:10.1371/journal.pmed.1004001)
Supplement: S6 Table — (PDF) [file pmed.1004001.s007.pdf]

S6 Table. Unadjusted and additionally adjusted models for the association between school exposure to noise intermittency ratio (IR, per 10% change) and 12-month change of working memory, complex working memory and inattentiveness (n = 2680 children, 9984 repeats).

Working memory (2-back number stimuli (detectability: d')) [a]						
Model	IR, street		IR, playground		IR, indoor	
	β (95%CI)	p-value	β (95%CI)	p-value	β (95%CI)	p-value
M0	0.73 (-1.32, 2.78)	0.486	-2.29 (-5.22, 0.64)	0.126	-2.19 (-4.65, 0.27)	0.080
M1	0.63 (-1.38, 2.63)	0.540	-2.56 (-5.43, 0.30)	0.079	-2.24 (-4.64, 0.16)	0.068
M2	0.71 (-1.29, 2.71)	0.486	-2.59 (-5.45, 0.27)	0.075	-2.27 (-4.67, 0.13)	0.063
M3	0.71 (-1.29, 2.71)	0.488	-2.59 (-5.44, 0.27)	0.076	-2.26 (-4.66, 0.14)	0.065
M4	0.84 (-1.17, 2.86)	0.411	-2.57 (-5.48, 0.35)	0.084	-2.19 (-4.64, 0.27)	0.081
M5	0.61 (-1.40, 2.61)	0.554	-2.42 (-5.31, 0.47)	0.100	-2.10 (-4.53, 0.34)	0.092
M6	0.89 (-1.13, 2.91)	0.388	-2.56 (-5.47, 0.35)	0.085	-2.17 (-4.62, 0.28)	0.083
M7	0.53 (-1.51, 2.57)	0.610	-3.34 (-6.23, -0.44)	0.024	-2.62 (-5.06, -0.19)	0.035
M8	0.79 (-1.24, 2.83)	0.445	-2.64 (-5.61, 0.34)	0.082	-2.22 (-4.70, 0.27)	0.080
M9	0.66 (-1.35, 2.66)	0.521	-2.44 (-5.33, 0.46)	0.099	-2.24 (-4.67, 0.20)	0.072
M10	0.76 (-1.25, 2.77)	0.459	-2.38 (-5.28, 0.52)	0.108	-2.13 (-4.57, 0.31)	0.087
M11	1.04 (-0.99, 3.08)	0.316	-2.26 (-5.20, 0.67)	0.130	-2.16 (-4.62, 0.31)	0.086
M12	0.45 (-1.59, 2.49)	0.667	-2.77 (-5.70, 0.16)	0.064	-2.47 (-4.94, 0.00)	0.050
M13	0.54 (-1.50, 2.58)	0.604	-2.60 (-5.53, 0.34)	0.083	-2.46 (-4.93, 0.01)	0.051
M14	0.69 (-1.33, 2.70)	0.504	-2.48 (-5.37, 0.42)	0.094	-2.44 (-4.87, 0.00)	0.050
M15	0.77 (-1.23, 2.76)	0.452	-2.67 (-5.52, 0.18)	0.066	-2.29 (-4.69, 0.10)	0.060
M16	0.77 (-1.23, 2.78)	0.450	-2.45 (-5.33, 0.43)	0.095	-2.22 (-4.72, 0.28)	0.081
M17	0.68 (-1.30, 2.66)	0.499	-2.39 (-5.25, 0.46)	0.101	-1.69 (-4.10, 0.72)	0.170
M18	0.71 (-1.29, 2.71)	0.486	-2.59 (-5.45, 0.27)	0.075	-2.27 (-4.67, 0.13)	0.063

S6 Table (Continued).

Complex working memory (3-back number stimuli (detectability:d')) [a]						
Model	IR, street		IR, playground		IR, indoor	
	β (95%CI)	p-value	β (95%CI)	p-value	β (95%CI)	p-value
M0	-0.63 (-2.25, 0.99)	0.446	-3.34 (-5.66, -1.02)	0.005	-2.76 (-4.70, -0.82)	0.005
M1	-0.67 (-2.26, 0.93)	0.412	-3.42 (-5.71, -1.13)	0.003	-2.76 (-4.66, -0.85)	0.005
M2	-0.66 (-2.25, 0.94)	0.419	-3.42 (-5.71, -1.13)	0.003	-2.76 (-4.66, -0.85)	0.005
M3	-0.66 (-2.25, 0.94)	0.418	-3.42 (-5.70, -1.13)	0.003	-2.74 (-4.64, -0.83)	0.005
M4	-0.67 (-2.28, 0.94)	0.413	-3.31 (-5.64, -0.98)	0.005	-2.54 (-4.49, -0.59)	0.011
M5	-0.57 (-2.17, 1.03)	0.485	-3.07 (-5.38, -0.75)	0.009	-2.44 (-4.37, -0.50)	0.014
M6	-0.80 (-2.42, 0.81)	0.329	-3.26 (-5.59, -0.94)	0.006	-2.57 (-4.52, -0.62)	0.010
M7	-0.68 (-2.31, 0.96)	0.417	-3.78 (-6.11, -1.45)	0.001	-3.06 (-5.00, -1.11)	0.002
M8	-0.59 (-2.22, 1.04)	0.476	-3.47 (-5.85, -1.08)	0.004	-2.74 (-4.72, -0.76)	0.007
M9	-0.75 (-2.35, 0.85)	0.357	-3.13 (-5.44, -0.81)	0.008	-2.56 (-4.49, -0.63)	0.009
M10	-0.68 (-2.28, 0.92)	0.405	-2.97 (-5.29, -0.64)	0.012	-2.43 (-4.37, -0.50)	0.014
M11	-0.43 (-2.05, 1.20)	0.607	-3.07 (-5.43, -0.72)	0.010	-2.56 (-4.52, -0.59)	0.011
M12	-0.70 (-2.32, 0.93)	0.400	-3.22 (-5.57, -0.88)	0.007	-2.68 (-4.64, -0.72)	0.007
M13	-0.66 (-2.29, 0.97)	0.428	-3.16 (-5.51, -0.81)	0.008	-2.52 (-4.49, -0.56)	0.012
M14	-0.67 (-2.27, 0.93)	0.414	-3.02 (-5.34, -0.69)	0.011	-2.77 (-4.70, -0.83)	0.005
M15	-0.57 (-2.17, 1.02)	0.480	-3.47 (-5.75, -1.19)	0.003	-2.76 (-4.66, -0.86)	0.004
M16	-0.57 (-2.17, 1.03)	0.485	-3.32 (-5.62, -1.02)	0.005	-2.76 (-4.75, -0.78)	0.006
M17	-0.76 (-2.35, 0.82)	0.345	-3.12 (-5.42, -0.82)	0.008	-2.31 (-4.24, -0.39)	0.019
M18	-0.66 (-2.25, 0.94)	0.419	-3.42 (-5.71, -1.13)	0.003	-2.76 (-4.66, -0.85)	0.005

S6 Table (Continued).

Model	IR, street		IR, playground		IR, indoor	
	β (95%CI)	p-value	β (95%CI)	p-value	β (95%CI)	p-value
M0	0.28 (-1.18, 1.74)	0.704	3.76 (1.67, 5.84)	<0.001	3.09 (1.35, 4.83)	<0.001
M1	0.44 (-0.99, 1.88)	0.544	3.77 (1.72, 5.82)	<0.001	3.05 (1.34, 4.76)	<0.001
M2	0.43 (-1.00, 1.87)	0.554	3.76 (1.71, 5.81)	<0.001	3.05 (1.34, 4.76)	<0.001
M3	0.44 (-1.00, 1.87)	0.551	3.76 (1.71, 5.81)	<0.001	3.03 (1.32, 4.74)	<0.001
M4	0.48 (-0.96, 1.92)	0.517	3.54 (1.46, 5.63)	<0.001	2.91 (1.16, 4.65)	0.001
M5	0.50 (-0.94, 1.94)	0.498	3.41 (1.33, 5.48)	0.001	2.87 (1.13, 4.60)	0.001
M6	0.47 (-0.97, 1.92)	0.522	3.63 (1.56, 5.71)	<0.001	3.02 (1.28, 4.76)	<0.001
M7	0.61 (-0.86, 2.07)	0.417	3.93 (1.84, 6.02)	<0.001	3.15 (1.41, 4.88)	<0.001
M8	0.35 (-1.12, 1.81)	0.643	3.44 (1.31, 5.57)	0.002	2.72 (0.95, 4.48)	0.003
M9	0.50 (-0.94, 1.94)	0.497	3.53 (1.46, 5.61)	<0.001	2.88 (1.15, 4.62)	0.001
M10	0.53 (-0.92, 1.97)	0.475	3.65 (1.57, 5.74)	<0.001	2.86 (1.12, 4.60)	0.001
M11	0.26 (-1.20, 1.72)	0.729	3.62 (1.52, 5.72)	<0.001	2.83 (1.07, 4.58)	0.002
M12	0.25 (-1.21, 1.71)	0.735	3.63 (1.52, 5.73)	<0.001	2.91 (1.15, 4.67)	0.001
M13	0.35 (-1.11, 1.81)	0.635	3.57 (1.47, 5.67)	<0.001	2.89 (1.14, 4.65)	0.001
M14	0.30 (-1.15, 1.74)	0.685	3.53 (1.44, 5.61)	<0.001	2.98 (1.24, 4.71)	<0.001
M15	0.42 (-1.01, 1.85)	0.565	3.78 (1.73, 5.82)	<0.001	3.05 (1.34, 4.76)	<0.001
M16	0.20 (-1.24, 1.63)	0.789	3.37 (1.31, 5.43)	0.001	2.38 (0.60, 4.16)	0.009
M17	0.55 (-0.86, 1.97)	0.444	3.40 (1.35, 5.46)	0.001	2.55 (0.83, 4.26)	0.004
M18	0.43 (-1.00, 1.86)	0.557	3.75 (1.70, 5.80)	<0.001	3.00 (1.30, 4.71)	<0.001

[a] A higher value in the test indicates better working memory; [b] a higher value in the test indicates greater inattentiveness. M0: Linear mixed models for the unadjusted change (i.e. adjusted for age, corresponding noise indicator × age, with child and school as nested random effects). M1 (Main adjustment set without TRAPs): M0 further adjusted for age, sex, maternal education, socio-economical vulnerability index at home. M2 (Main model): M1 further adjusted for outdoor or indoor traffic-related air pollution (TRAPs) at school, respectively, for models with the corresponding outdoor or indoor noise indicators. M3 to M17 correspond to M2 further adjusted for: M3: type of school, M4: Paternal education, M5: Foreign origin, M6: Marital status, M7: Overweight, M8: Computer games in the weekend, M9: Siblings, M10: Adoption, M11: Smoking during pregnancy, M12: Preterm birth, M13: Birth weight, M14: Breastfeeding, M15: Socio-economical vulnerability index at school, M16: School education quality, M17: Behavioural problems, M18: Paired school by design as nested random effect.
