# Supplementary material for: Exposure to road traffic noise and cognitive development in schoolchildren in Barcelona, Spain: A population-based cohort study
Source: PLoS Med. 2022 Jun 2;19(6):e1004001. doi: 10.1371/journal.pmed.1004001 (PMC9162347; doi:10.1371/journal.pmed.1004001)
Supplement: S7 Table — (PDF) [file pmed.1004001.s008.pdf]

S7 Table. Unadjusted and additionally adjusted models for the association between school exposure to number of noise events (NE, per 50 events) and 12-month change of working memory, complex working memory and inattentiveness (n = 2680 children, 9984 repeats).

**Working memory (2-back number stimuli (detectability: d'))** [a]						
Model	NE, street		NE, playground		NE, indoor	
	β (95%CI)	p-value	β (95%CI)	p-value	β (95%CI)	p-value
M0	-4.27 (-7.06, -1.49)	0.003	-2.28 (-5.37, 0.81)	0.147	-2.67 (-5.48, 0.14)	0.062
M1	-4.46 (-7.17, -1.74)	0.001	-2.53 (-5.56, 0.51)	0.103	-2.71 (-5.45, 0.03)	0.052
M2	-4.38 (-7.08, -1.67)	0.002	-2.68 (-5.71, 0.35)	0.082	-2.72 (-5.45, 0.01)	0.051
M3	-4.37 (-7.07, -1.66)	0.002	-2.68 (-5.71, 0.35)	0.083	-2.72 (-5.46, 0.02)	0.051
M4	-4.51 (-7.26, -1.75)	0.001	-2.66 (-5.72, 0.41)	0.089	-2.86 (-5.64, -0.08)	0.044
M5	-4.83 (-7.56, -2.09)	<0.001	-2.73 (-5.77, 0.31)	0.079	-2.62 (-5.37, 0.13)	0.062
M6	-4.54 (-7.29, -1.78)	0.001	-2.65 (-5.71, 0.41)	0.089	-2.72 (-5.49, 0.04)	0.054
M7	-4.33 (-7.10, -1.56)	0.002	-2.98 (-6.05, 0.09)	0.057	-3.07 (-5.85, -0.30)	0.030
M8	-4.59 (-7.41, -1.77)	0.001	-2.98 (-6.12, 0.15)	0.062	-2.88 (-5.68, -0.07)	0.044
M9	-4.58 (-7.31, -1.84)	0.001	-2.57 (-5.61, 0.48)	0.099	-2.66 (-5.41, 0.09)	0.058
M10	-4.80 (-7.54, -2.06)	<0.001	-2.56 (-5.61, 0.50)	0.101	-2.61 (-5.37, 0.14)	0.063
M11	-4.60 (-7.36, -1.84)	0.001	-2.52 (-5.60, 0.56)	0.108	-2.47 (-5.26, 0.31)	0.082
M12	-5.27 (-8.04, -2.49)	<0.001	-2.90 (-5.98, 0.19)	0.066	-2.84 (-5.63, -0.05)	0.046
M13	-4.83 (-7.60, -2.06)	<0.001	-2.42 (-5.51, 0.67)	0.125	-2.88 (-5.67, -0.10)	0.042
M14	-4.75 (-7.48, -2.01)	<0.001	-2.57 (-5.62, 0.49)	0.099	-2.86 (-5.61, -0.11)	0.042
M15	-4.41 (-7.11, -1.72)	0.001	-2.75 (-5.78, 0.28)	0.075	-2.77 (-5.49, -0.05)	0.046
M16	-4.52 (-7.24, -1.79)	0.001	-2.63 (-5.70, 0.45)	0.094	-2.72 (-5.61, 0.18)	0.066
M17	-4.55 (-7.26, -1.84)	<0.001	-2.73 (-5.74, 0.28)	0.075	-2.17 (-4.89, 0.55)	0.118
M18	-4.38 (-7.08, -1.67)	0.002	-2.68 (-5.71, 0.35)	0.082	-2.72 (-5.45, 0.01)	0.051

S7 Table (Continued).

Complex working memory (3-back number stimuli (detectability: d')) [a]						
Model	NE, street		NE, playground		NE, indoor	
	β (95%CI)	p-value	β (95%CI)	p-value	β (95%CI)	p-value
M0	-3.93 (-6.14, -1.73)	<0.001	-2.91 (-5.36, -0.46)	0.020	-3.21 (-5.43, -1.00)	0.005
M1	-4.00 (-6.16, -1.83)	<0.001	-3.03 (-5.46, -0.61)	0.014	-3.22 (-5.39, -1.04)	0.004
M2	-3.99 (-6.16, -1.82)	<0.001	-3.03 (-5.46, -0.61)	0.014	-3.22 (-5.39, -1.04)	0.004
M3	-3.96 (-6.13, -1.79)	<0.001	-3.03 (-5.45, -0.61)	0.014	-3.18 (-5.36, -1.00)	0.004
M4	-4.33 (-6.53, -2.13)	<0.001	-2.99 (-5.44, -0.53)	0.017	-3.01 (-5.22, -0.79)	0.008
M5	-4.34 (-6.53, -2.15)	<0.001	-2.81 (-5.24, -0.38)	0.024	-2.94 (-5.14, -0.75)	0.009
M6	-4.43 (-6.63, -2.23)	<0.001	-2.90 (-5.34, -0.45)	0.020	-3.04 (-5.24, -0.84)	0.007
M7	-4.10 (-6.33, -1.88)	<0.001	-3.18 (-5.65, -0.72)	0.011	-3.56 (-5.78, -1.34)	0.002
M8	-4.72 (-6.98, -2.47)	<0.001	-3.18 (-5.69, -0.67)	0.013	-3.39 (-5.63, -1.14)	0.003
M9	-4.32 (-6.50, -2.13)	<0.001	-2.82 (-5.25, -0.39)	0.023	-3.02 (-5.21, -0.83)	0.007
M10	-4.38 (-6.57, -2.19)	<0.001	-2.77 (-5.21, -0.33)	0.026	-2.91 (-5.11, -0.72)	0.009
M11	-4.37 (-6.58, -2.16)	<0.001	-2.83 (-5.29, -0.36)	0.025	-2.99 (-5.21, -0.76)	0.009
M12	-4.54 (-6.75, -2.32)	<0.001	-2.77 (-5.23, -0.31)	0.027	-3.03 (-5.25, -0.81)	0.007
M13	-4.23 (-6.45, -2.01)	<0.001	-2.82 (-5.29, -0.35)	0.026	-2.99 (-5.22, -0.77)	0.008
M14	-4.33 (-6.52, -2.15)	<0.001	-2.61 (-5.05, -0.17)	0.036	-3.22 (-5.41, -1.03)	0.004
M15	-3.97 (-6.13, -1.81)	<0.001	-3.08 (-5.50, -0.66)	0.013	-3.26 (-5.43, -1.09)	0.003
M16	-4.12 (-6.29, -1.95)	<0.001	-3.07 (-5.53, -0.61)	0.014	-3.34 (-5.65, -1.03)	0.005
M17	-4.28 (-6.46, -2.10)	<0.001	-2.87 (-5.29, -0.45)	0.020	-2.80 (-4.98, -0.62)	0.012
M18	-3.99 (-6.16, -1.82)	<0.001	-3.03 (-5.46, -0.61)	0.014	-3.22 (-5.39, -1.04)	0.004

S7 Table (Continued).

Inattentiveness (Attention Network Test, Hit reaction time standard error, HRT-SE, ms) [b]						
Model	NE, street		NE, playground		NE, indoor	
	β (95%CI)	p-value	β (95%CI)	p-value	β (95%CI)	p-value
M0	2.24 (0.25, 4.23)	0.027	2.31 (0.12, 4.49)	0.038	3.32 (1.32, 5.31)	0.001
M1	2.15 (0.20, 4.10)	0.031	2.21 (0.06, 4.36)	0.044	3.17 (1.21, 5.12)	0.002
M2	2.13 (0.18, 4.08)	0.032	2.22 (0.07, 4.37)	0.043	3.15 (1.20, 5.11)	0.002
M3	2.10 (0.14, 4.05)	0.035	2.21 (0.06, 4.36)	0.044	3.13 (1.17, 5.08)	0.002
M4	2.33 (0.35, 4.31)	0.021	2.10 (-0.07, 4.27)	0.058	3.05 (1.06, 5.03)	0.003
M5	2.43 (0.45, 4.41)	0.016	1.97 (-0.20, 4.13)	0.075	3.01 (1.03, 4.98)	0.003
M6	2.43 (0.45, 4.41)	0.016	2.13 (-0.04, 4.30)	0.054	3.11 (1.14, 5.09)	0.002
M7	2.41 (0.41, 4.41)	0.018	2.35 (0.15, 4.54)	0.036	3.31 (1.32, 5.30)	0.001
M8	2.42 (0.39, 4.45)	0.020	1.88 (-0.34, 4.11)	0.097	2.89 (0.88, 4.90)	0.005
M9	2.38 (0.41, 4.35)	0.018	2.04 (-0.13, 4.20)	0.065	2.99 (1.01, 4.96)	0.003
M10	2.54 (0.56, 4.52)	0.012	2.09 (-0.08, 4.26)	0.059	2.97 (0.99, 4.95)	0.003
M11	2.58 (0.59, 4.58)	0.011	2.25 (0.07, 4.43)	0.043	3.06 (1.07, 5.06)	0.003
M12	2.52 (0.52, 4.52)	0.013	2.22 (0.03, 4.41)	0.047	3.22 (1.22, 5.22)	0.002
M13	2.39 (0.39, 4.39)	0.019	2.12 (-0.07, 4.31)	0.058	3.24 (1.25, 5.24)	0.001
M14	2.33 (0.35, 4.31)	0.021	2.13 (-0.04, 4.31)	0.054	3.14 (1.16, 5.11)	0.002
M15	2.12 (0.17, 4.07)	0.033	2.22 (0.07, 4.37)	0.043	3.15 (1.20, 5.11)	0.002
M16	1.89 (-0.07, 3.85)	0.058	1.71 (-0.47, 3.89)	0.125	2.24 (0.16, 4.31)	0.035
M17	2.17 (0.22, 4.13)	0.029	1.90 (-0.24, 4.04)	0.082	2.64 (0.69, 4.59)	0.008
M18	2.12 (0.17, 4.07)	0.034	2.21 (0.06, 4.36)	0.044	3.14 (1.18, 5.09)	0.002

[a] A higher value in the test indicates better working memory; [b] a higher value in the test indicates greater inattentiveness. M0: Linear mixed models for the unadjusted change (i.e. adjusted for age, corresponding noise indicator × age, with child and school as nested random effects). M1 (Main adjustment set without TRAPs): M0 further adjusted for age, sex, maternal education, socio-economical vulnerability index at home. M2 (Main model): M1 further adjusted for outdoor or indoor traffic-related air pollution (TRAPs) at school, respectively, for models with the corresponding outdoor or indoor noise indicators. M3 to M17 correspond to M2 further adjusted for: M3: type of school, M4: Paternal education, M5: Foreign origin, M6: Marital status, M7: Overweight, M8: Computer games in the weekend, M9: Siblings, M10: Adoption, M11: Smoking during pregnancy, M12: Preterm birth, M13: Birth weight, M14: Breastfeeding, M15: Socio-economical vulnerability index at school, M16: School education quality, M17: Behavioural problems, M18: Paired school by design as nested random effect.
