# Supplementary material for: Exposure to road traffic noise and cognitive development in schoolchildren in Barcelona, Spain: A population-based cohort study
Source: PLoS Med. 2022 Jun 2;19(6):e1004001. doi: 10.1371/journal.pmed.1004001 (PMC9162347; doi:10.1371/journal.pmed.1004001)
Supplement: S1 Text — (PDF) [file pmed.1004001.s009.pdf]

S1 Text

Estimation of the individual indoor noise levels in the classroom

We derived predictive models to obtain noise levels in classrooms using multivariable linear regression models. The dependent variable was the measured indoor level of road traffic noise. The independent variables of the final model accounted for the floor level (ground or first, second, third or higher floor), room orientation (classroom oriented towards: indoor area, outdoor courtyard, or directly to the street), outdoor levels of road traffic noise, type of courtyard (open, semi-open, built) and an interaction between the outdoor levels of road traffic noise and room orientation. Other tested variables not included in the final model were the distance from the classroom to the street, type of window (good sealing versus bad sealing) and average daily traffic intensity.

Firstly, we carried out univariate analysis of all variables. We evaluated their normal distribution, assessed the linearity of the relationship between the dependent variable and all potential predictors, and considered variable transformation, accordingly. Second, we introduced the variables consecutively into the multivariate model in order of highest to lowest R^2, provided that they added more than 1% to the R^2 and kept the expected direction in the association. We then applied backward regression, retaining variables with a p-value of < 0.1.

Regression diagnostics tests included normality of residuals, homoscedasticity, and influential data points. We assessed model performance with leave-one-out cross-validation (LOOCV), obtaining three measures of goodness-of-fit: the LOOCV R2 (pseudo R2, the square of the coefficient of correlation between the predicted and observed values of noise levels), LOOCV RMSE (the Root Mean Square Error: the standard deviation of the residuals), and LOOCV MAE (the Mean Absolute Error: the average of the absolute values of the residuals). Importantly, we calculated the next performance criteria: i) the Fraction Bias (FB), which accounts for the over- or underestimation of the model, with an ideal value of zero, ii) the Normalized Mean Square Error (NMSE), which accounts for the scatter of the model and accounts both for systematic and random error, with an ideal value of zero, and iii) the Factor of 2 (FAC2), defined as the ratio of model predictions to observed values that are ≥ 0.5 and ≤ 2. The model was considered acceptable if FAC2 > 0.5, $|FB| < 0.3$, and NMSE < 1.5. Finally, we predicted levels of indoor noise for all the classrooms using the parameter estimates derived from the regression model. The model had an Adjusted R^2 of 67.7% with an LOOCV R^2 of 54.8%, RMSE of 3.6 and MAE of 2.9. The performance criteria were widely met, with an FB and NMSE close to the ideal zero value (respectively, -2.601e-09 and 0.004) and FAC2 = 1.
